# Real-time six-dimensional spatiotemporal tracking of single anisotropic nanoparticles in live cells by integrated multifunctional light-sheet nanoscopy

**DOI:** 10.1007/s00604-023-05633-1

**Published:** 2023-01-16

**Authors:** Yingying Cao, Seungah Lee, Kyungsoo Kim, Jong-Young Kwak, Seong Ho Kang

**Affiliations:** 1 Department of Chemistry, Graduate School, Kyung Hee University, Yongin-Si, Gyeonggi-Do 17104 Republic of Korea; 2 Department of Applied Chemistry and Institute of Natural Sciences, Kyung Hee University, Yongin-Si, Gyeonggi-Do 17104 Republic of Korea; 3 Department of Applied Mathematics, Kyung Hee University, Yongin-Si, Gyeonggi-Do 17104 Republic of Korea; 4 Department of Pharmacology, Ajou University School of Medicine, 164 World Cup-Ro, Yeongtong-Gu, Suwon, 16499 Republic of Korea

**Keywords:** Six-dimensional tracking, Anisotropic nanoparticle, Multifunctional light-sheet nanoscopy, Single living cell, Bioimaging, Fucoidan

## Abstract

An integrated multifunctional light-sheet nanoscopy (*i*MLSN) combined with differential interference contrast, total internal reflection, epifluorescence, a super-resolution radial fluctuation-stream module, and a wavelength-dependent light sheet was developed to simultaneously realize the six-dimensional (6D) vector-valued (three coordinates + rotational dynamics (azimuth and elevation angles) + transport speed) tracking of anisotropic nanoparticles in single living cells. The wavelength-dependent asymmetric scattering of light by gold nanorods was used to trigger signals depending on the polarizer angle, and real-time photo-switching was achieved by turning the polarizer, obtaining a series of super-resolution images, and tracking using different polarization directions and two channels. This technique was employed to directly observe native gold nanorods (AuNRs; 5 nm diameter × 15 nm length) and surface-functionalized AuNRs during their endocytosis and transport at the upper and attaching side membrane regions of single living cells, revealing that the AuNRs bound to the membrane receptors. The nanorods were subsequently internalized and transported away from the original entry spots. Detailed dynamic information regarding the rotation properties and endocytosis speed during the transmembrane process was also acquired for each region. The developed technique can be considered useful for the real-time monitoring of intracellular transport at various regions in single living cells, as well as for 6D vector-valued non-fluorescence super-resolution imaging and tracking.

**Graphical Abstract:**

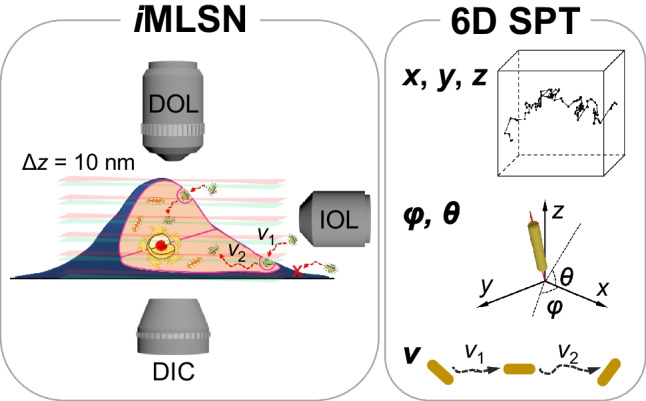

**Supplementary Information:**

The online version contains supplementary material available at 10.1007/s00604-023-05633-1.

## Introduction

Single-particle tracking (SPT) has been applied to numerous biological applications due to its ability to elucidate the dynamics of complex intracellular processes, such as the uptake and transport of pharmaceuticals [1], virus-cell interactions [2], and working mechanisms of motor proteins [3]. Various biological processes in living cells are usually accompanied by translational and rotational movements [4]. In particular, the SPT technique was combined with surface-functionalized gold nanorods (AuNRs) exhibiting excellent photostability [5], high biocompatibility [6], and desirable anisotropic scattering properties [4] to realize dynamic tracking in engineered environments and living cells. Such a technique can be applied in dark-field (DF) microscopy [7], total internal reflection (TIR) scattering microscopy [8], differential interference contrast (DIC) microscopy [9], and light-sheet (LS) microscopy [10]. For example, DF microscopy has been used to track the transmembrane process of peptide-modified gold nanoparticles in three dimensions [11] and capture the rotation and orientation dynamics of individual AuNRs at the cell sidewall [7]. The main challenge associated with DF microscopy is the distinguishment between the individual optical probes and the cellular organelles in a crowded cellular environment [12]. Another common technique for live cell imaging is TIR microscopy [2], which reduces the background noise and enhances the image contrast. However, TIR microscopy can only detect events taking place within the evanescent field (i.e., ~ 200 nm) [2]. Alternatively, parallax-DIC microscopy has been developed to track single AuNRs over five dimensions (three-dimensional (3D) coordinates plus azimuth and elevation angles) for elucidating the dynamics of intracellular cargo transport [9]. However, when the size of the imaging probe is reduced, detection by DIC imaging becomes more challenging [13].

AuNR tracking probes measuring at least several tens of nanometers (i.e., diameters > 25 nm) are often used in SPT studies and have been reported to exhibit significant effects on cellular uptake, cell dynamics, and intracellular transport [14, 15]. Furthermore, since the intracellular mobility of labeled target molecules depends on the size of the label [16], small imaging probes are required for the labeling or tracking of biomolecules in complex cellular environments. As a result, the dynamic tracking of small imaging probes in living cells continues to attract growing attention in the field of SPT. Regarding the detection of tiny plasmonic nanoparticles, photothermal imaging utilizing the absorption cross-sections of plasmonic nanoparticles could track 5-nm antibody-modified gold nanoparticles in living cells [17]. However, the applicability of this method on cell imaging is limited by the potential disruption of biological specimens caused by the high-power laser beam required for photothermal imaging and the heat generated upon nanoparticle irradiation [4].

To overcome these limitations, we modified the light-sheet-based microscopy to develop multifunctional LS nanoscopy. The LS was focused using an illumination objective lens which excited the scattering of plasmonic nanoparticles to a maximum, thus improving the ability to detect tiny plasmonic nanoparticles. Furthermore, the LS could be adjusted to illuminate the distinct regions of cells with thin optical sectioning [18], which increased the imaging depth of microscopy and produced good signal-to-noise ratios as well as less photobleaching and phototoxicity.

Herein, we report the development of an *i*MLSN system and method for spatiotemporal 6D vector-valued SPT of tiny native (5 nm in diameter) and functionalized AuNRs (i.e., fucoidan-conjugated AuNRs (Fu-AuNRs)) during transmembrane and intracellular transport, at various regions of single living cells. The developed technique significantly increased the potential for real-time detection and tracking of the superlocalization and motional behavior of tiny single nanoparticles at various regions of living cells. In addition, *i*MLSN could achieve long-time live-cell imaging with an excellent signal-to-noise ratio, owing to the thin LS illumination. The entire transmembrane and intracellular transport processes of single imaging probes have been revealed with high accuracy using the *i*MLSN technique.

## Materials and methods

### Materials

Native AuNRs and cetyltrimethylammonium bromide–capped AuNRs (CTAB-AuNRs) were purchased from Nanopartz (Salt Lake City, UT, USA). AuNRs of different sizes were used in this work, i.e., 5-nm AuNRs (5 nm diameter × 15 nm length), 25-nm AuNRs (25 nm diameter × 90 nm length), and 40-nm AuNRs (40 nm diameter × 80 nm length). Fucoidan from *Fucus vesiculosus*, dynasore hydrate, polyvinyl alcohol (PVA), glycerol, agarose, poly-*L*-lysine solution (PLL, 0.01% w/v in H_2_O), and trypan blue solution (0.4%) were purchased from Sigma-Aldrich (St. Louis, MO, USA). Roswell Park Memorial Institute Medium (RPMI-1640) was purchased from GenDEPOT (Barker, TX, USA). Dulbecco’s phosphate buffered saline (DPBS) was purchased from HyClone Laboratories (Logan, UT, USA). MitoTracker Green (*E*_x_/*E*_m_ = 490/516 nm) and Hoechst 33342 (*E*_x_/*E*_m_ = 350/461 nm) for cell staining were purchased from Invitrogen (Carlsbad, CA).

### 6D *i*MLSN system

**Fig. 1:**
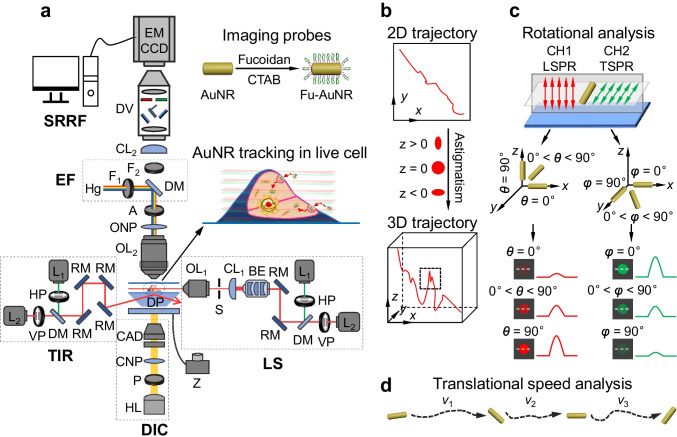
Proposed 6D integrated multifunctional light-sheet nanoscopy and spatiotemporal single-particle tracking approach. **a** Schematic of the *i*MLSN system used for AuNR tracking in live cells. **b** Use of astigmatism imaging to superlocalize the AuNR trajectory in three dimensions (*x*, *y*, *z*). **c** Combinatorial approach using *p*-pol (red) and *s-*pol (green) lasers to calculate the azimuth (*φ*) and elevation (*θ*) angles. **d** Use of translational speed analysis to determine the instantaneous speed (*v*) of AuNRs during the 6D spatiotemporal tracking process

The 6D *i*MLSN system (Fig. 1 and Fig. S1) was developed at our laboratory based on our previously reported prism-based 3D integrated light-sheet super-resolution microscopy system (Fig. 1a) [19, 20]. The 6D *i*MLSN system was combined with LS, TIR, epifluorescence (EF), DIC, and super-resolution radial fluctuations (SRRF-stream) modules, and the dimensions of the generated light-sheets were measured and analyzed [21] (see the “Supplementary information” for details).

### Preparation and characterization of Fu-AuNRs

The conjugation of fucoidan on the AuNR surface (Fu-AuNRs) was conducted following a previously reported method [22] based on the electrostatic interaction between the cationic –N^+^(CH_3_)_3_ group of CTAB and the anionic sulfate group of fucoidan (see the “Supplementary information” for details).

For the imaging of the AuNRs embedded onto solidified PVA, a PVA solution (20 mL) was prepared following a previously described protocol [23]. The AuNRs were fixed onto solidified PVA to investigate the scattering intensity bias from the chromatic aberrations of the illumination objective lens (see the “Supplementary information” for details).

In addition, the AuNRs and Fu-AuNRs were characterized by transmission electron microscopy (TEM, 2100F, JEOL Ltd, Tokyo, Japan) [24], UV–vis spectroscopy (MultiSpec-1501, Shimadzu, Tokyo, Japan), dynamic light scattering (802DLS, Viscotek, Westborough, MA, USA), and Fourier transform infrared (FT-IR) spectroscopy (Perkin Elmer, Inc., Norwalk, CT, USA) [19] (see the “Supplementary information” for details).

### Cell culture for single-particle tracking in live cells

Human lung cancer cells (A549) placed on a coverslip were incubated with native AuNRs and Fu-AuNRs for 1 h, followed by the staining of cell mitochondria and nucleus. Finally, the cell coverslip was subjected to 6D *i*MLSN for image acquisition (see the “Supplementary information” for details).

### Analysis of spatiotemporal single-particle tracking data

The obtained raw images were preprocessed using ImageJ (NIH) and MATLAB (Mathworks, Torrance, CA, USA) software. The trajectories (*x-*, *y-*, and *z*-coordinates) of single AuNRs were superlocalized in three dimensions using the astigmatism method (see “Supplementary information” for details) [21].

## Results and discussion

### Integrated multifunctional light-sheet nanoscopy

The 6D *i*MLSN system (Fig. 1 and Fig. S1) possesses a number of desirable properties, including ability to achieve cell imaging through super-resolution radial fluctuations (SRRF-stream) and DIC microscopy, dual-wavelength detection, astigmatism imaging, and superlocalization. Based on the experimental requirements, different setup combinations could be selected. Table 1 shows the limitations of different imaging techniques applied in SPT field. In the experiments, AuNRs were used as the imaging probes, and their journey inside single living cells was monitored in real time. The generated two light sheets (a thickness of ~ 1.4 µm and a length of ~ 11.5 µm for the 698 nm laser and a thickness of ~ 1.2 µm and a length of ~ 10 µm for the 532 nm laser; Fig. S2) were smaller than the height of the cancer cell layer (e.g., A549 cells, ~ 4–6 μm) [25]. Thus, only parts of the single living cells were illuminated, which reduced the background and photodamage during live cell imaging. AuNRs with lengths of several tens of nanometers were clearly distinguished by DIC and scattering analysis (Fig. S3a–f). As previously reported, when the size of the AuNRs is reduced, their detection by DIC microscopy becomes challenging [13]. Indeed, the smaller 5-nm native AuNRs (5 nm diameter × 15 nm length) showed poor image contrast in the DIC image, while the longitudinal surface plasmon resonance (LSPR) and transverse surface plasmon resonance (TSPR) scattering images of the excited AuNRs exhibited good signal-to-noise ratios (Fig. S3g–l). Therefore, *i*MLSN was able to detect the tiny 5-nm AuNRs on coverslips (Fig. S3) and track random translational movement of tiny AuNRs in 100% glycerol (Movie S1).

**Table 1:** Comparison of multidimensional SPT techniques

Imaging technique	Probe size (*D* × *L*, nm)	Application	Limitations	Reference
DF microscopy	43 × 84	Direct observation of the orientation dynamics of single anisotropic nanoparticles	Difficult to distinguish between the individual optical probes and the cellular organelles in a crowded cellular environment	[7]
TIR microscopy	25 × 60	Simultaneous translational and rotational imaging with *s-* and *p-*polarized light	Only appropriate for detecting the events taking place within the evanescent field	[8]
DIC microscopy	40 × 80	Elucidating the dynamics of intracellular cargo transport by 5D SPT	Small nanoparticles are nearly invisible	[9]
SNaPT	5	Tracking individual 5-nm gold nanoparticles in live cells	Potential disruption of biological specimens by the high-power laser beam	[17]
*i*MLSN	5 × 15	Real-time 6D SPT of single tiny anisotropic nanoparticles in live cells	Resolve the rotation angle in the range of 0–90°	This work

*D*, diameter; *L*, length; *DF*, dark field; *TIR*, total internal reflection; *DIC*, differential interference contrast; *SNaPT*, single nanoparticle photothermal tracking; *iMLSN*, integrated multifunctional light-sheet nanoscopy

Astigmatism-based methods are common techniques in 3D super-resolution imaging [21]. Here, we inserted a cylindrical lens (*f* = 1,000 mm) into the imaging pathway to change the orientation and ellipticity of the image point spread function (PSF) as the *z*-position varies (Fig. 1b). To localize the *z*-coordinates of biomolecule-conjugated AuNRs, we constructed a calibration curve of the PSF widths by scanning immobilized fucoidan-conjugated AuNR (5 nm Fu-AuNRs) at *z*-intervals of 10 nm (Fig. S4a). The center position (*z* = 0 nm) was defined as the average focal plane in which the PSF width in the *x*-direction (*w*_*x*_) equaled that in the *y*-direction (*w*_*y*_). The PSF became ellipsoidal along the *x-* and *y*-axes when it was below and above the average focal plane, respectively (Fig. S4b). After comparison of the *w*_*x*_ and *w*_*y*_ values of the experimental PSF with those of the calibration curves, the obtained *z*-positions of the AuNRs were corrected due to a refractive index mismatch [21].

Given that many biological processes involve the rotational movement of cargo [4], we tracked the in-plane and out-of-plane rotational dynamics of the AuNRs by investigating the effects of excitation laser polarization direction and AuNR orientation (transverse vs. longitudinal) on the polarization properties. Specifically, a short-wavelength horizontally polarized (*s*-pol) laser and a long-wavelength vertically polarized (*p*-pol) laser were selected to induce TSPR and LSPR in the AuNRs (Fig. 1c). The in-plane or out-of-plane rotation of the AuNRs resulted in periodic changes in the scattering intensity due to the effects of TSPR and LSPR. A dual-view (DV) device allowed these scattering intensity changes to be simultaneously recorded by an electron-multiplying cooled charge-coupled device (EMCCD) camera [19] and enabled the motion trajectory and resolutions of the in-plane and out-of-plane rotational dynamics to be determined. To monitor the rotation and orientation of the 5-nm AuNRs, we used 532-nm *s*-pol and 698-nm *p*-pol light sheets to excite the TSPR and LSPR, respectively. As shown in Fig. 1c, the LSPR (CH1) scattering intensity increased upon the rotation of a flat-lying AuNR to a vertical position. When the longitudinal axis of the AuNR was parallel to the polarization direction of the 532-nm *s*-pol laser, the TSPR was minimally excited to yield the weakest scattering intensity (CH2), whereas this intensity was maximized when the longitudinal axis was perpendicular to the polarization direction of the laser.

The instantaneous speed (*v*) of the AuNRs was calculated during the transmembrane process (Fig. 1d) and then used to examine the motional modes of the AuNRs that were caused by AuNR − cell interactions. Fluorescence staining is commonly employed to understand the intracellular transport and distribution of target molecules within a cell; thus, epifluorescence (EF)-based SRRF-stream detection was used to reconstruct the raw fluorescence images [26]. Because fucoidan exhibits an anticancer effect and can interact with mitochondria [27], the mitochondria and nucleus of a single living cell were stained using the MitoTracker Green and Hoechst 33342 fluorescence dyes, respectively. The morphology of a single live A549 cell was imaged by the DIC and EF modes (Fig. S5a–c). Following the SRRF-stream technique, the locations of the mitochondria and nucleus could be defined more precisely than in the corresponding raw fluorescence images (Fig. S5d). This combined technique thus permits the intracellular dynamics and localization of an imaging probe to be analyzed more accurately in a single living cell. The adopted approach also enabled simultaneous tracking with 6D vectors, namely, the spatial trajectory (*x*, *y*, *z*), instantaneous speed (*v*), and rotational behavior (*φ* and *θ*) of the anisotropic plasmonic AuNRs during their transmembrane and intracellular transport.

### Functionalization of AuNRs for single-particle tracking

Nanorods are useful in the context of live cell imaging owing to their small size, which results in fewer morphological defects, shorter membrane wrapping time, and enhanced cell internalization [15, 28]. Because surface ligands and charges are known to affect the cell-nanoprobe interactions [15], the morphology, size, and optical/surface properties of native AuNRs, CTAB-AuNRs, and Fu-AuNRs were probed using TEM, UV–vis absorbance spectroscopy, FT-IR spectroscopy, and zeta potential measurements (Fig. S6). The conjugation of fucoidan on the AuNR surface was achieved via electrostatic interactions between the cationic –N^+^(CH_3_)_3_ group of CTAB and the anionic sulfate group of fucoidan. Fu-AuNRs exhibited a rod-like morphology (5 nm diameter × 15 nm length) as well as TSPR and LSPR absorption peaks at 520 and 730 nm, respectively. Moreover, after conjugation with fucoidan, the zeta potential of AuNRs became negative (− 24.7 mV for Fu-AuNRs) (see the “Supplementary information” for details). Similarly to sulfated polysaccharides, fucoidan contains abundant *L*-fucose and sulfate ester groups (Fig. S6a) and exhibits antitumor, antiviral, and anticancer properties [27]. Thus, Fu-AuNRs are considered as drug carriers whose SPT imaging is valuable for revealing the internalization process of drug carriers.

### Characterization of AuNRs using the *i*MLSN system for single-particle scattering

Because the scattering intensity from a AuNR depends on its relative orientation and the local power density of the light sheet [8], individual AuNRs fixed on a transparent polymer (i.e., polyvinyl alcohol, PVA) were used to confirm whether an offset caused by the chromatic aberration of the illumination objective lens took place. As a proof of concept, the individual 5-nm native AuNRs fixed on solidified PVA were observed by 6D *i*MLSN (Fig. S7). We found that the LSPR and TSPR scattering intensities of the AuNRs illuminated by both 532- and 698-nm light-sheets increased only slightly compared with those of the AuNRs illuminated by any single light sheet (Fig. S7e and f). These results indicate that the bias from the chromatic aberration of the illumination lens is negligible when calculating the AuNR orientation, which can be attributed to the low illumination powers of the light sheets, the DV device, and the corresponding band-pass filters that are specific to the LSPR and TSPR scatterings of the AuNRs.

To immobilize Fu-AuNRs on coverslips, clean coverslips were immersed in a PLL solution for 30 min. After washing with deionized water (three times) and drying at 25 ℃ for 2 h, the coverslips were coated with PLL to promote the adsorption of the negatively charged nanoparticles. The localization precision of the *i*MLSN system was evaluated by immobilizing Fu-AuNRs on a PLL-coated coverslip for multiple frames at an exposure of 10 ms. The *x-*, *y-*, and *z*-coordinates obtained for each frame afforded a 3D location distribution, with standard deviations (*σ*) of 2.9, 2.7, and 6.2 nm in the *x*-, *y*-, and *z*-directions, respectively (Fig. S8a). The corresponding full width at half maximum values (FWHM = 2.35*σ*) in the above directions equaled 6.8, 6.3, and 14.6 nm, respectively [11]. We also evaluated the localization of the individual Fu-AuNRs in live cell (Fig. S8b). The standard deviations (*σ*) of the localization distributions in the *x-*, *y-*, and *z*-directions were slightly larger than those of the AuNRs fixed on the PLL-coated coverslip, which could be due to fluctuation of the distributions within the live cells. These results indicate the ability of *i*MLSN to localize fixed AuNRs and the individual Fu-AuNRs present in single living cells in a highly precise manner.

In addition, Fu-AuNRs were immobilized on a PLL-coated scratched coverslip to confirm the accuracy of our imaging system for superlocalizing the positions of motorial nanoprobes (Fig. S9). The *i*MLSN stage motion was controlled in the *z*-direction using a *z*-motor, and in the *x-* and *y*-directions by manual operation. After superlocalizing the 3D coordinates of the AuNRs during the *i*MLSN stage motion, the average distance between two AuNRs was calculated (Fig. S9c–e). All calculated distances were comparable with the measured distance between NRs (i.e., ~ 6.04 μm), indicating that our imaging system could accurately superlocalize the positions of motorial AuNRs.

### Deciphering the motion of various AuNRs by 6D *i*MLSN

**Fig. 2:**
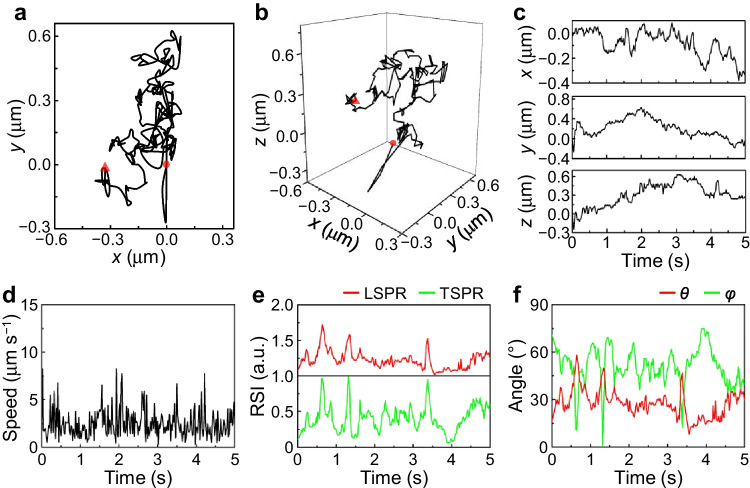
Deciphering the real-time spatiotemporal SPT motion of a 5 nm Fu-AuNR in 100% glycerol using 6D *i*MLSN. **a** 2D and **b** 3D trajectories of a single Fu-AuNR, and **c** its corresponding displacements along the *x-*, *y-*, and *z*-axes. **d** Instantaneous speed, **e** normalized intensities, and **f** calculated angles of a single Fu-AuNR as a function of time. The red dot and triangle denote the start and end points of movement, respectively

The anisotropic and plasmonic AuNRs are widely used to track both spatial movement and rotation [4]. The motions of 5 nm native and Fu-AuNR species were tracked in 100% glycerol (Fig. 2 and Fig. S10). It was revealed that the high viscosity of glycerol [29] limited the movement of Fu-AuNRs within the range of micrometers [10], thereby resulting in narrower translational diffusion ranges compared with those of native AuNRs (Fig. S10). The native AuNRs exhibited stronger fluctuations in the *x-*, *y*-, and *z*-directions (Fig. S10c), while Fu-AuNRs exhibited smaller *x-*, *y-,* and *z*-displacement ranges (Fig. 2c). The mean squared displacements (MSDs) of the trajectories of the native AuNRs and Fu-AuNRs were calculated to determine the AuNR motion type. The MSDs were linearly proportional to the log time (Fig. S11), thereby suggesting the occurrence of Brownian diffusion. The gradient was steeper for the native AuNRs, indicating the restricted movement of Fu-AuNRs in glycerol. The corresponding diffusion coefficients (*D*_c_), which represent the freedom of Brownian motion, were estimated to be 0.095 and 0.018 μm^2^ s^−1^ for the native AuNRs and Fu-AuNRs, respectively. The native AuNRs (16.9 ± 15.4 µm s^−1^) exhibited a higher average instantaneous speed than Fu-AuNRs (2.8 ± 1.7 µm s^−1^), result that is in agreement with their 3D trajectories and displacements (Fig. 2d and Fig. S10d). Moreover, the movement speeds of the native AuNRs and Fu-AuNRs exhibited intense heterogeneity owing to their random movement in glycerol [30].

The rotational behavior of the AuNRs was also restricted by the high viscosity of glycerol, as revealed by their normalized TSPR and LSPR scattering intensity traces. More specifically, the scattering intensities varied with random on–off bursts, indicating the occurrence of random in-plane and out-of-plane rotations. However, the scattering intensities and flickering frequencies of the native AuNRs differed considerably from those of Fu-AuNRs. The native AuNRs exhibited faster TSPR and LSPR intensity fluctuations between the maximum and minimum values, whereas the Fu-AuNRs exhibited slower and less intense fluctuations (Fig. 2e and Fig. S10e). The corresponding *φ* and *θ* values were calculated and fast and wide-angle rotation was evident for the native AuNRs (Fig. S10f). After modification with fucoidan, the rotational behavior of AuNRs became even slower, especially the out-of-plane rotation (elevation angle, *θ*, Fig. 2f). The differences in movement and rotation between these species were attributed to the interactions between the AuNR surface and surrounding solution. The long molecular chain and abundant hydrophilic functional groups of fucoidan [31] can increase the frictional force and numbers of hydrophilic interactions and van der Waals forces between the AuNRs and glycerol molecules [10]. Surface modification increased the weight and volume of the AuNRs and reduced the movement speeds of the modified derivates, which could also contribute to the distinctly different motions of the different species. Based on these results, the native AuNRs are expected to move more freely in glycerol, followed by the Fu-AuNRs. Overall, our results indicate that the developed 6D vector-valued *i*MLSN system is suitable for studying the movement and rotational behavior of surface-modified tiny anisotropic nanoparticles in solution.

### Real-time motion of AuNRs at the plasma membrane with dynasore-mediated inhibition

The wavelength-dependent super-resolution-based *i*MLSN technique was used to collect different sample information by variation in the detection mode employed. For example, LS microscopy detected the imaging probes in whole cells by optical *z*-sectioning with a thin light-sheet. More specifically, Fu-AuNRs at different *z*-heights within the micrometer range were observed in A549, RAW264.7, and HeLa cells by LS nanoscopy (Fig. S12a–d), while Fu-AuNRs with *z*-heights within the hundred nanometer range (i.e., the evanescent field layer) were observed by TIR microscopy (Fig. S12e–h). These results indicate that the *i*MLSN technique allowed tracking of the cell internalization process of various single AuNRs on the upper part and attaching side of single living cell membranes. Furthermore, the effect of the size of AuNRs on the cellular uptake was investigated (Fig. S13). It was revealed that 5 × 15 nm Fu-AuNRs were internalized to a range of 2 − 3 times larger than 40 × 80 nm and 25 × 90 nm Fu-AuNRs, due to the shorter membrane wrapping time required for 5 × 15 nm Fu-AuNRs [15]. This result shows that tiny imaging probes are favorable for cellular uptake and can minimize the interference with target molecules [14]. Therefore, the 5 × 15 nm AuNRs were used as imaging probes for further study.

According to previous reports, cells take up Fu-AuNRs in the culture medium via receptor-mediated endocytosis [10, 27]. This finding was confirmed by incubating A549 cells with Fu-AuNRs under normal conditions (37 ℃ and 5% CO_2_, control), at 4 °C, and in the presence of dynasore hydrate (80 µM), which was introduced to inhibit receptor-mediated endocytosis [32]. Compared with the control, the uptake of Fu-AuNRs was reduced by half when the A549 cells were incubated at 4 °C (Fig. S14). Furthermore, when the A549 cells were treated with dynasore hydrate, the uptake of Fu-AuNR was 4.5 times lower than that of the control experiment, thereby confirming that the uptake of Fu-AuNRs was dependent on the incubation temperature and endocytosis process [9]. Furthermore, the single-particle motional behaviors of the 5 nm Fu-AuNRs at different membrane regions of the single living cells were observed in the presence of dynasore hydrate (Figs. S15 and S16). When Fu-AuNRs bind to the cell membrane, they gradually lose their translational and rotational behaviors and tend to rotate in-plane [9]. After incubating dynasore hydrate-treated A549 cells with the Fu-AuNRs for 4 h, the NRs adhered to the upper part and attaching side of the cell membrane (Figs. S15a–c and S16a–c). The motional behaviors of the Fu-AuNRs were monitored over 1 h, and images were acquired every 20 min. The Fu-AuNRs showed lateral diffusion within a small region in addition to rapid intensity fluctuations (Movie S2 and Movie S3), thereby indicating their simultaneous in-plane and out-of-plane rotation at the upper part and attaching side of the cell membrane. The Fu-AuNRs remained bound to the cell membrane after 4 h of incubation, although those present on the upper part and attaching side maintained their fast in-plane and out-of-plane rotation without any apparent loss in rotational freedom over a period of 1 h (Figs. S15d and S16d). These results indicated that the Fu-AuNRs present in the culture media could not be endocytosed by the live cells under dynasore hydrate treatment, thereby confirming the mechanism of receptor-mediated endocytosis.

### Spatiotemporal tracking of the AuNRs in single living cells by 6D *i*MLSN

**Fig. 3:**
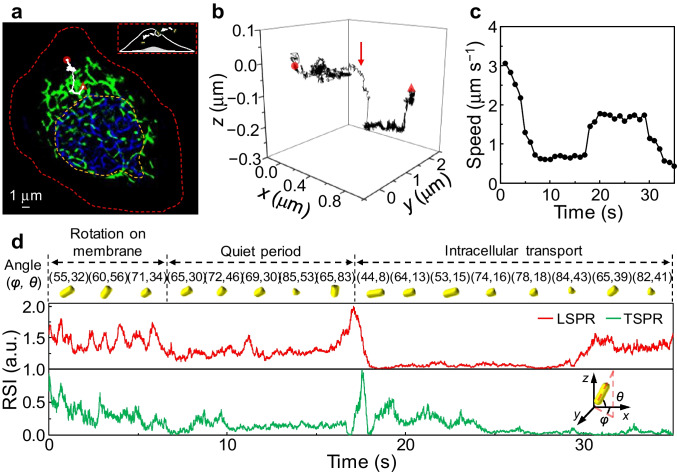
Representative single 5-nm Fu-AuNR 6D spatiotemporal tracking on the upper membrane of a single living A549 cell. **a** EF-based SRRF image of a single A549 cell with a stained nucleus (blue) and mitochondria (green). (Inset) 2D tracking trajectory of Fu-AuNR indicated by the white line. The red- and yellow-dotted lines indicate the contours of the cell and nucleus, respectively. The red circle and triangle denote the start and end points of movement, respectively. **b** 3D tracking trajectory of a Fu-AuNR highlighted in the white line in (**a**). The red arrow indicates the internalization of the Fu-AuNR. **c** and **d** Corresponding speeds and descriptions of the different rotational states of a single Fu-AuNR on the upper membrane of a single live A549 cell

We initially tracked the motion of various AuNRs during the transmembrane process at the upper cell membrane after incubating A549 cells with the 5 nm native AuNRs and 5 nm Fu-AuNRs for 1 h (Fig. 3, Fig. S17, Movie S4). Given that the negative surface charge of the cell hindered AuNR attachment to the cell membrane, the various AuNRs exhibited a range of motions and rotations. Because of the weak negative charge of the native AuNR surface, active lateral movements were observed (Fig. S17a–c) in addition to in-plane and out-of-plane rotation (Fig. S17d). The negatively charged Fu-AuNRs initially tended to undergo active translational movement (Fig. 3a-c) and out-of-plane rotation on the cell membrane (Fig. 3d). The various AuNRs gradually lost their translational and rotational freedoms, and movement essentially stopped temporarily for the native AuNRs (Fig. S17d) and Fu-AuNRs (Fig. 3d). This period with limited movement and active rotation corresponded to the assembly of endocytic vesicles around the AuNRs. These results demonstrate that the surface functional groups and surface charges of imaging probes affect their motional behaviors on the cell membrane [33].

At the end of the quiet periods, the AuNRs clearly exhibited translational motion in the *z*-direction (red arrows, Fig. 3b and Fig. S17b), indicating their cell internalization. Notably, the Fu-AuNRs exhibited an out-of-plane angle of 83° at the end of the quiet period, thus suggesting that internalization took place with the longitudinal axis of the NRs almost perpendicular to the upper part of the cell membrane. This result indicates that fucoidan binds to the cell receptors [27], although electrostatic repulsion also exists between the negatively charged cell membrane and negatively charged Fu-AuNRs, ultimately resulting in uptake with a high out-of-plane angle. After the corresponding quiet periods, the active lateral movements and rotations were restored, indicating that the AuNRs had been transported into the cells. Different rotational motions were observed for the two species during intracellular transport. As can be seen in Figure S17d, the native AuNRs rotated rapidly with large in-plane and out-of-plane angles and the Fu-AuNRs exhibited a slow orientational change without active rotation (Fig. 3d). These different motional phenomena could be attributed to the sizes and surface modifications of the AuNRs. We note here that these 5-nm AuNR species are significantly smaller than the diameters of the early endosomes (50–100 nm) [34]. Furthermore, the Fu-AuNRs were tightly bound to the membrane receptors, resulting in a weak rotational freedom with respect to the early endosomes.

**Fig. 4:**
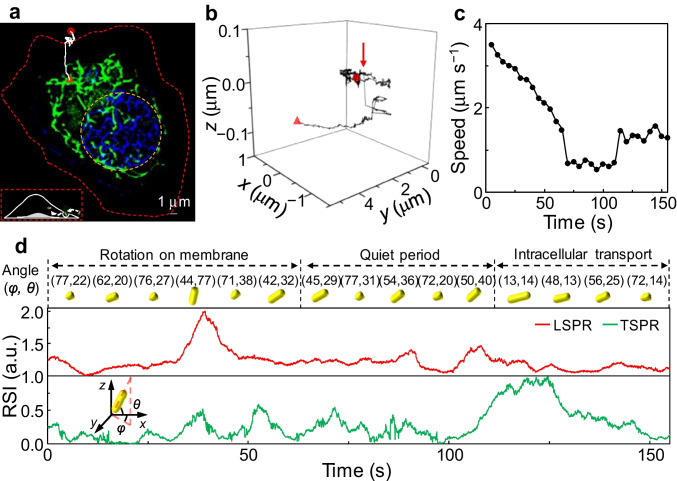
Representative single 5 nm Fu-AuNR 6D spatiotemporal tracking on the attaching side membrane of a single living A549 cell. **a** EF-based SRRF image of a single A549 cell with a stained nucleus (blue) and mitochondria (green). (Inset) 2D tracking trajectory of Fu-AuNR indicated by the white line. The red- and yellow-dotted lines indicate the contours of the cell and nucleus, respectively. The red circle and triangle denote the start and end points of movement, respectively. **b** 3D tracking trajectory of a Fu-AuNR highlighted in the white line in (**a**). The red arrow indicates the internalization of the Fu-AuNR. **c** and **d** Corresponding speeds and descriptions of the different rotational states of a single Fu-AuNR on the attaching side membrane of a single living A549 cell

The motions of the Fu-AuNRs at the attaching side of the A549 cell membrane (Fig. 4 and Movie S5) differed from those at the upper part of the membrane because of the obstruction of the coverslip and extracellular matrix [18]. More specifically, the Fu-AuNRs at the attaching side tended to exhibit active translational movement (Fig. 4a-c) and slower out-of-plane and in-plane rotations on the cell membrane (Fig. 4d). After a quiet period of ~ 48 s (Fig. 4d), the Fu-AuNR was internalized through the attaching side of the cell membrane, as indicated by a clear translational motion in the *z*-direction (red arrow, Fig. 4b). At the moment of endocytosis, the Fu-AuNR exhibited an out-of-plane angle of 28° and an in-plane angle of 32° (Fig. 4d), indicating that the NR longitudinal axis was almost perpendicular to the attaching side during endocytosis. This result is similar to that observed at the upper part of the cell membrane, which suggests that negatively charged biomolecule-conjugated AuNRs tend to adopt a perpendicular longitudinal axis in relation to the cell membrane during endocytosis. Moreover, the Fu-AuNR underwent a slow orientation change without active rotation during its intracellular transport (Fig. 4d). In particular, the DIC images of A549 cells at the initial and end stages of SPT imaging using *i*MLSN showed that long-term imaging as well as the biocompatibility of Fu-AuNRs and fluorescent dyes of mitochondria and nucleus did not affect the survival of the cells (Fig. S18).

Four additional SPT images of 5 nm AuNRs in single live A549 cells were acquired by 6D *i*MLSN and summarized (Fig. S19). Native AuNRs showed fast translational speed and rotational movement due to the weak interaction between native AuNRs and cell membrane. With the surface modification of fucoidan, the interactions were increased significantly because of fucoidan-receptor binding and high viscosity of cell membrane [7]. Thus, Fu-AuNRs moved and rotated slowly on cell membrane. At a certain point of time, the movement speed and rotation of AuNRs slowed down and a relatively quiet period was observed for all AuNRs. During this period, the AuNRs showed minimal speeds and slow orientation changes instead of active rotation, indicating that AuNRs were enclosed in cell membrane. The AuNRs with different surface modifications (native and Fu-AuNRs) at various regions of cell membrane (upper part and attaching side) exhibited different quiet durations and orientation angles to cell membrane. Therefore, the developed *i*MLSN can be considered useful for the real-time monitoring of tiny imaging probes in single living cells, as well as for 6D vector-valued non-fluorescence super-resolution imaging and tracking.

## Conclusion

We developed an *i*MLSN system and method to track the motion and rotation of single 5 nm AuNRs with 6D vectors and decipher the motional and rotational behaviors of cargos in living cells at a subdiffraction-limit resolution. Such a resolution is significantly different to the ones achieved by highly inclined and laminated optical sheet microscopy [35] and other conventional light-sheet microscopy [18]. The developed *i*MLSN significantly improved the detectability of tiny (5 nm in diameter) plasmonic nanoparticles in living cells. The real-time 6D vector-valued SPTs of tiny native and surface-functionalized AuNRs at various regions of single living cells were studied using the *i*MLSN technique. It was revealed that the surface modification of imaging probes and endocytosis sites at different regions of single living cells affected their motional and rotational behaviors during endocytosis and intracellular transport. Despite some limitations, the developed *i*MLSN technique provided detailed dynamic information related to the rotation properties and endocytosis speeds during the transmembrane process at different membrane regions of single living cells. Therefore, this method is suitable for long real-time monitoring of drug delivery cargos, virus infections, and relevant bioimaging fields, exhibiting less photobleaching and phototoxicity issues related to fluorescence dyes.

## Supplementary Information

Below is the link to the electronic supplementary material.

Supplementary file1 (PDF 2953 KB)

Supplementary file2. Detection of native AuNRs (5 nm diameter × 15 nm length) in 100% glycerol by the iMLSM system. The AuNRs were simultaneously illuminated by 698 and 532 nm light sheets (MP4 578 KB)

Supplementary file3. Motion and scattering intensity of Fu-AuNRs on a cell membrane at the upper part of a single live cell upon treatment with dynasore hydrate (MP4 4088 KB)

Supplementary file4. Motion and scattering intensity of Fu-AuNRs on a cell membrane at the attaching side of a single live cell upon treatment with dynasore hydrate (MP4 4428 KB)

Supplementary file5. (left) SPT imaging of 5 nm Fu-AuNRs on a cell membrane at the upper part of a single live A549 cell. (right) Corresponding changes in the scattering intensity of the 5 nm Fu-AuNRs in single live A549 cells for CH1 and CH2 (MP4 5337 KB)

Supplementary file6. (left) SPT imaging of 5 nm Fu-AuNRs on a cell membrane at the attaching side of a single live A549 cell. (right) Corresponding changes in the scattering intensity of the 5 nm Fu-AuNRs in single live A549 cells for CH1 and CH2 (MP4 3476 KB)

## Data Availability

All data needed to evaluate the conclusions in the paper are present in the paper and/or the “Supplementary information.”

## References

[1] Ruthardt N, Lamb DC, Bräuchle C (2011). Single-particle tracking as a quantitative microscopy-based approach to unravel cell entry mechanisms of viruses and pharmaceutical nanoparticles. Mol Ther.

[2] Liu S-L, Wang Z-G, Xie H-Y, Liu A-A, Lamb DC, Pang D-W (2020). Single-virus tracking: from imaging methodologies to virological applications. Chem Rev.

[3] Kural C, Kim H, Syed S, Goshima G, Gelfand VI, Selvin PR (2005). Kinesin and dynein move a peroxisome in vivo: a tug-of-war or coordinated movement?. Sci.

[4] Gu Y, Ha JW, Augspurger AE, Chen K, Zhu S, Fang N (2013). Single particle orientation and rotational tracking (SPORT) in biophysical studies. Nanoscale.

[5] Chakkarapani SK, Zhang P, Ahn S, Kang SH (2016). Total internal reflection plasmonic scattering-based fluorescence-free nanoimmunosensor probe for ultra-sensitive detection of cancer antigen 125. Biosens Bioelectron.

[6] Murphy CJ, Gole AM, Stone JW, Sisco PN, Alkilany AM, Goldsmith EC, Baxter SC (2008). Gold nanoparticles in biology: beyond toxicity to cellular imaging. Acc Chem Res.

[7] Xu D, He Y, Yeung ES (2014). Direct imaging of transmembrane dynamics of single nanoparticles with darkfield microscopy: improved orientation tracking at cell sidewall. Anal Chem.

[8] Marchuk K, Ha JW, Fang N (2013). Three-dimensional high-resolution rotational tracking with superlocalization reveals conformations of surface-bound anisotropic nanoparticles. Nano Lett.

[9] Chen K, Gu Y, Sun W, Bin D, Wang G, Fan X, Xia T, Fang N (2017). Characteristic rotational behaviors of rod-shaped cargo revealed by automated five-dimensional single particle tracking. Nat Commun.

[10] Xiao L, Qiao Y, He Y, Yeung ES (2011). Imaging translational and rotational diffusion of single anisotropic nanoparticles with planar illumination microscopy. J Am Chem Soc.

[11] Shen H, Xiong B, Xu R, Cheng X, He Y, Yeung ES (2014). 3D darkfield imaging and single particle tracking of peptide-coated nanocargoes in live cells. Anal Methods.

[12] Xiao L, Ha JW, Wei L, Wang G, Fang N (2012). Determining the full three-dimensional orientation of single anisotropic nanoparticles by differential interference contrast microscopy. Angew Chem Int Ed.

[13] Wu Y, Ali MRK, Chen K, Fang N, El-Sayed MA (2019). Gold nanoparticles in biological optical imaging. Nano Today.

[14] Cognet L, Leduc C, Lounis B (2014). Advances in live-cell single-particle tracking and dynamic super-resolution imaging. Curr Opin Chem Biol.

[15] Verma A, Stellacci F (2010). Effect of surface properties on nanoparticle-cell interactions. Small.

[16] Chou LYT, Ming K, Chan WCW (2011). Strategies for the intracellular delivery of nanoparticles. Chem Soc Rev.

[17] Lasne D, Blab GA, Berciaud S, Heine M, Groc L, Choquet D, Cognet L, Lounis B (2006). Single nanoparticle photothermal tracking (SNaPT) of 5-nm gold beads in live cells. Biophys J.

[18] Wäldchen F, Schlegel J, Götz R, Luciano M, Schnermann M, Doose S, Sauer M (2020). Whole-cell imaging of plasma membrane receptors by 3D lattice light-sheet *d*STORM. Nat Commun.

[19] Chakkarapani SK, Sun Y, Kang SH (2019). Ultrasensitive norovirus nanoimmunosensor based on concurrent axial super-localization of ellipsoidal point spread function by 3D light sheet microscopy. Sens Actuators B Chem.

[20] Chakkarapani SK, Sun Y, Lee S, Fang N, Kang SH (2018). Three-dimensional orientation of anisotropic plasmonic aggregates at intracellular nuclear indentation sites by integrated light sheet super-resolution microscopy. ACS Nano.

[21] Huang B, Wang W, Bates M, Zhuang X (2008). Three-dimensional super-resolution imaging by stochastic optical reconstruction microscopy. Sci.

[22] Manivasagan P, Bharathiraja S, Santha Moorthy M, Oh Y-O, Song K, Seo H, Oh J (2017). Anti-EGFR antibody conjugation of fucoidan-coated gold nanorods as novel photothermal ablation agents for cancer therapy. ACS Appl Mater Interfaces.

[23] Stoenescu S, Packirisamy M, Truong V-V (2013). Improved alignment of gold nanorods embedded in polymer films. IJTAN.

[24] Zhang P, Lee S, Yu H, Fang N, Kang SH (2015). Super-resolution of fluorescence-free plasmonic nanoparticles using enhanced dark-field illumination based on wavelength-modulation. Sci Rep.

[25] Tang M, Wang Y, Tang D, Xiu P, Yang Z, Chen Y, Wang H (2021). Influence of the PM2.5 water-soluble compound on the biophysical properties of A549 cells. Langmuir.

[26] Culley S, Tosheva KL, Matos Pereira P, Henriques R (2018). SRRF: Universal live-cell super-resolution microscopy. Int J Biochem Cell Biol.

[27] Hsu H-Y, Hwang P-A (2019). Clinical applications of fucoidan in translational medicine for adjuvant cancer therapy. Clin Transl Med.

[28] Salatin S, Maleki Dizaj S, Yari Khosroushahi A (2015). Effect of the surface modification, size, and shape on cellular uptake of nanoparticles. Cell Biol Int.

[29] Ferreira AGM, Egas APV, Fonseca IMA, Costa AC, Abreu DC, Lobo LQ (2017). The viscosity of glycerol. J Chem Thermodyn.

[30] Zheng H, Claridge SA, Minor AM, Alivisatos AP, Dahmen U (2009). Nanocrystal diffusion in a liquid thin film observed by in situ transmission electron microscopy. Nano Lett.

[31] Oliveira C, Granja S, Neves NM, Reis RL, Baltazar F, Silva TH, Martins A (2019). Fucoidan from Fucus vesiculosus inhibits new blood vessel formation and breast tumor growth in vivo. Carbohydr Polym.

[32] Jiang Y, Huo S, Mizuhara T, Das R, Lee Y-W, Hou S, Moyano DF, Duncan B, Liang X-J, Rotello VM (2015). The interplay of size and surface functionality on the cellular uptake of sub-10 nm gold nanoparticles. ACS Nano.

[33] Gu Y, Sun W, Wang G, Fang N (2011). Single particle orientation and rotation tracking discloses distinctive rotational dynamics of drug delivery vectors on live cell membranes. J Am Chem Soc.

[34] Murk JLAN, Humbel BM, Ziese U, Griffith JM, Posthuma G, Slot JW, Koster AJ, Verkleij AJ, Geuze HJ, Kleijmeer MJ (2003). Endosomal compartmentalization in three dimensions: Implications for membrane fusion. Proc Natl Acad Sci USA.

[35] Tokunaga M, Imamoto N, Sakata-Sogawa K (2008). Highly inclined thin illumination enables clear single-molecule imaging in cells. Nat Methods.

