# Supplementary material for: Real-time six-dimensional spatiotemporal tracking of single anisotropic nanoparticles in live cells by integrated multifunctional light-sheet nanoscopy
Source: Mikrochim Acta. 2023 Jan 16;190(2):54. doi: 10.1007/s00604-023-05633-1 (PMC9841004; doi:10.1007/s00604-023-05633-1)
Supplement: Supplementary file 1 — Supplementary file1 (PDF 2953 KB) [file 604_2023_5633_MOESM1_ESM.pdf]

Electronic Supplementary Material

Real-time six-dimensional spatiotemporal tracking of single anisotropic nanoparticles in live cells by integrated multifunctional light-sheet nanoscopy

Yingying Cao[1], Seungah Lee[2], Kyungsoo Kim[3], Jong-Young Kwak[4], Seong Ho Kang[1,2*]

[1] Department of Chemistry, Graduate School, Kyung Hee University, Yongin-si, Gyeonggi-do 17104, Republic of Korea

[2] Department of Applied Chemistry and Institute of Natural Sciences, Kyung Hee University, Yongin-si, Gyeonggi-do 17104, Republic of Korea

[3] Department of Applied Mathematics, Kyung Hee University, Yongin-si, Gyeonggi-do 17104, Republic of Korea

[4] Department of Pharmacology, Ajou University School of Medicine, 164 World cup-ro, Yeongtong-gu, Suwon 16499, Republic of Korea

*Correspondence: shkang@khu.ac.kr

Contents

1.1 Abbreviations -- S3
1.2 6D *i*MLSN system and measurements of light sheet dimensions ------------------------------ S4
1.3 Preparation of Fu-AuNRs and AuNRs embedded in solidified PVA ------------------------- S6
1.4 Sample preparation for characterization ---S7
1.5 Cell culture for single-particle tracking in live cells --- S7
1.6 Analysis of spatiotemporal single-particle tracking data --------------------------------------- S8
2.1 Functionalization of the AuNRs for single-particle tracking --------------------------------- S11
Fig. S1 -- S12
Fig. S2 -- S13
Fig. S3 -- S14
Fig. S4 -- S15
Fig. S5 -- S16
Fig. S6 -- S17
Fig. S7 -- S18
Fig. S8 -- S19
Fig. S9 -- S20
Fig. S10--- S21
Fig. S11--- S22
Fig. S12--- S23
Fig. S13--- S24
Fig. S14--- S25
Fig. S15--- S26
Fig. S16--- S27
Fig. S17--- S28
Fig. S18--- S29
Fig. S19--- S30
Movie files (MP4) -- S31

1 Supporting materials and methods

1.1 Abbreviations

L_1, 532 nm laser; L_2, 698 nm laser; HP, horizontal polarizer; VP, vertical polarizer; DM, dichroic mirror; RM, reflecting mirror; BE, beam expander; CL_1, cylindrical lens (f = 500 mm); S, slit; OL_1, objective lens for illumination; HL, halogen lamp; P, polarizer; CNP, condenser Nomarski prism; CAD, condenser aperture diaphragm; DP, dove-type prism; OL_2, objective lens for detection; ONP, objective Nomarski prism; A, analyzer; Hg, mercury lamp for EF mode; F_1, excitation filter for fluorescence; F_2, band-pass filter for fluorescence; CL_2, cylindrical lens (f = 1000 mm); DV, dual-view; EMCCD, electron-multiplying cooled charge-coupled device; Z, *z*-stage controller; CH1 and CH2, the two channels of the DV device; 6D, six-dimensional; AuNRs, gold nanorods; CTAB-AuNRs, cetyltrimethylammonium bromide-capped AuNRs; Fu-AuNRs, fucoidan-conjugated AuNRs; SPT, single-particle tracking; DF, dark field; TIR, total internal reflection; DIC, differential interference contrast; LS, light sheet; SNaPT, single nanoparticle photothermal tracking; *i*MLSN, integrated multifunctional light-sheet nanoscopy; EF, epifluorescence; SRRF, super-resolution radial fluctuation; PVA, polyvinyl alcohol; PLL, poly-*L*-lysine; RPMI-1640, Roswell Park Memorial Institute Medium; DPBS, Dulbecco's phosphate buffered saline; TEM, transmission electron microscope; FT-IR, Fourier-transform infrared; LSPR, longitudinal surface plasmon resonance; TSPR, transverse surface plasmon resonance; *s*-pol, horizontally polarized; *p*-pol, vertically polarized; RSI, relative scattering intensity; MSD, mean squared displacement; EFL, evanescent field layer.

1.2 6D *i*MLSN system and measurements of light sheet dimensions

An Olympus BX53 microscope was used to construct the imaging system for multi-dimensional SPT. When 5 nm AuNRs were used as the imaging probe, adjustable 698 nm (MRL-FN-698, 200 mW, Changchun New Industries Optoelectronics Tech. Co., Ltd., Changchun, China) and 532 nm (MGL-III-532, 250 mW, Changchun New Industries Optoelectronics Tech. Co., Ltd.) continuous-wave lasers were used as the excitation sources. A dichroic mirror (LWP-45-R488/532-T633-PW-1025-C, Korea) was used to adjust the two laser lines (L_1 and L_2). A rotatable polarizer (U-AN360P, Olympus Optical, Co., Ltd., Tokyo, Japan) was placed in front of each laser, *i.e.*, a horizontal polarized polarizer for the 532 nm laser and a vertical polarized polarizer for the 698 nm laser, to simultaneously acquire the *s*-pol and *p*-pol scattering images of the AuNRs. The laser directions were controlled by two mirrors (MM2-311-12.5, Semrock, Rochester, NY, USA). The light-sheet was generated by placing a beam expander (GBE02-A, SM1TC, Thorlabs Inc., Newton, NJ, USA), a cylindrical lens (CL_1) (LJ1144RM, f = 500 mm, Thorlabs), and a slit (VA100/M, Thorlabs) in the light path, and it was focused on the sample chamber using a 10× illumination objective lens (OL_1) (UPlanFLN, NA = 0.3, Olympus). The cell coverslip was fastened to a microdove-type prism (PS990, Thorlabs). All imaging signals were collected using a 100× detecting objective lens (OL_2) with an adjustable numerical aperture (UPlanFLN, iris, NA = 0.6–1.3, Olympus). A dual-view device (DV2, Photometrics, Tucson, AZ, USA) and a cylindrical lens (CL_2) (LJ1516RM, f = 1000 mm, Thorlabs) were mounted between the detection objective lens and electron-multiplying cooled charge-coupled device (EMCCD) camera (512 × 512-pixel imaging array, iXon Ultra, Andor, Belfast, UK). Benefitting from the DV2 device, the detected scattering signals were split into two parts based on their wavelengths and simultaneously recorded by an EMCCD camera. Thus, the TSPR scattering triggered by the *s*-pol 532 nm light-sheet (or TIR) and the LSPR

scattering triggered by the *p*-pol 698 nm light-sheet (or TIR) were acquired simultaneously. Two band-pass filters were inserted into the DV2 device, *i.e.*, a 520/15 nm filter (Semrock) for the short-wavelength slot and a 680/35 nm filter (Semrock) for the long-wavelength slot. The sample stage was controlled using a *z*-motor (LEP MAC 6000, LUDL Electronic Products Ltd., Hawthorne, NY, USA) with 10 nm intervals. The two polarized lasers were mounted on the other side of microscope and were focused via mirrors to produce TIR in the microdove-type prism under the detecting objective lens. DIC microscopy was used to confirm the locations of the cell outlines and organelles. The light emitted from the halogen lamp passed through the polarizer and condenser Nomarski prism to form two vibrating perpendicular rays that were made parallel by passing through the condenser aperture diaphragm (U-UCD8, Olympus). The split beams were passed through the specimen and were combined using an analyzer and a Nomarski prism (U-DICT, Olympus) to form DIC images. EF-based SRRF was employed to determine the location of the cellular mitochondria and nucleus. The light from a mercury (Hg) lamp was passed through an excitation filter and reflected by a dichroic mirror to excite the fluorescent dye in the live cells. A band-pass filter was used to collect the emitted fluorescent signals. The SRRF-stream mode was used to reconstruct the organelle locations by measuring the intensity gradient convergence of the fluorescence signals. All imaging data were acquired using MetaMorph software (Universal Imaging, Sunnyvale, CA, USA) with an exposure time of 10 ms. When 40 nm AuNRs were used as the imaging probes, the adjustable 698 nm laser was replaced with an adjustable 637 nm laser (MRL-III-637, 200 mW, Changchun New Industries Optoelectronics Tech. Co., Ltd.), and the corresponding band-pass filter in the long-wavelength slot of the DV2 device was changed to a 620/14 nm filter (Semrock). All other configurations were maintained.

For measurements of the light sheet dimensions, a flow chamber was prepared from two coverslips and double-sided tape. Two concentrated fluorescent sphere solutions (F8792, E_x/E_m = 540/560 nm; F8791, E_x/E_m = 715/755 nm; Invitrogen) were added to the flow chamber. The illumination objective lens was adjusted so that the light sheets passed through the flow chamber to excite the fluorescent spheres. The fluorescence signals were collected and imaged using an EMCCD camera. The cylindrical lens was rotated so that the light sheets became horizontal, which enabled the detection of their width and length. Rotating the cylindrical lens by 90° rendered the light sheets perpendicular, which allowed the determination of their corresponding thickness. The collected images were analyzed using ImageJ software (1.53a version, NIH, Bethesda, MD, USA).

1.3 Preparation of Fu-AuNRs and AuNRs embedded in solidified PVA

To prepare Fu-AuNRs, an aliquot (1 mL) of the CTAB-AuNR dispersion (5 nm, 25 nm or 40 nm) was centrifuged one time, the precipitated CTAB-AuNRs was redispersed in deionized water (1 mL), and the obtained dispersion was supplemented with a solution of fucoidan (10 mg) in deionized water (1 mL) and stirred at 24 °C for 24 h. The resulting surface-modified AuNRs were precipitated by centrifugation (13572 g, 30 min, three times), washed with DPBS (1 mL, three times) to remove any unbound fucoidan, and re-suspended in DPBS (1 mL).

To prepare the PVA solution, PVA powder (2.04 g) was added to deionized water (20 mL) and heated to 90 °C under stirring for 30 min to obtain a homogeneous solution. The PVA solution was cooled to 22 °C and glycerol (32 μL) was added gradually under stirring for 30 min. The resulting PVA solution was subjected to ultrasonication (42 kHz, 100 W, 10 min) to remove bubbles, and 100 μL of 5 nm AuNRs were mixed with the as-obtained PVA solution. After stirring for 20 min, the mixture was subjected to ultrasonication for 10 min, cast onto a 22 mm × 22 mm

coverslip, and then dried at 60 °C for 5 h. The concentration of the AuNR suspension used in the sample preparation was ~6.94×10^{12} particles mL^{-1}

1.4 Sample preparation for characterization

To acquire TEM images, 5 μL of AuNR suspension was added to a Cu-grid (carbon coated, Ted Pella, Inc., Redding, CA, USA), and the TEM images were acquired after the Cu-grid was dried. For FT-IR measurements, the AuNRs were dispersed in deionized water and 10 μL of the solution was placed onto the stage of the FT-IR spectrometer. The sample solution was dried for 10 min at room temperature, then background correction and sample testing were carried out to obtain the corresponding spectra. The UV-vis absorption spectra were acquired with the AuNR suspension injected into a quartz glass cell of 10 mm light path. For zeta potential measurements, the AuNR suspension was added to the zeta cell. Each sample was tested three time and its mean value was used in the present work. The concentration of the AuNR suspension used in all characterizations was ~6.94×10^{12} particles mL^{-1}.

1.5 Cell culture for single-particle tracking in live cells

A549 human lung cancer cells were purchased from the Korean Cell Line Bank (Seoul, Korea) and grown in RPMI-1640 supplemented with 10% fetal bovine serum, 100 unit mL^{-1} penicillin sulfate, and 100 μg mL^{-1} streptomycin inside a humidified incubator with 5% CO_2 at 37 °C. For the single-cell imaging experiments, the cells were seeded on a 22 mm × 22 mm coverslip (No. 1, Deckglaser, Freiburg, Germany) housed in a Petri dish and used after overnight incubation. Cell culture medium was used to dilute the native AuNRs, and Fu-AuNRs to ~1.5×10^9 particles mL^{-1} prior to cell incubation. The cells on the coverslips were immersed in the AuNR-containing cell

culture medium and incubated for 1 h. The cell mitochondria and nucleus were stained with MitoTracker Green (E_x/E_m = 490/516 nm) and Hoechst 33342 (E_x/E_m = 350/461 nm), respectively, for 0.5 h in a cell incubator according to the Invitrogen user guide. After incubation, the cell coverslip and substrate coverslip (18 mm × 18 mm) formed a chamber with two pieces of double-sided tape between them. The chamber was filled with the cell culture medium and the chamber sides were sealed with nail polish. Finally, the coverslips were placed on the prism and subjected to 6D *i*MLSN.

1.6 Analysis of spatiotemporal single-particle tracking data

The PSFs of the AuNRs in the tracking movies were analyzed using an elliptical Gaussian function to deduce the centroid coordinates (x_0 and y_0) and image widths (w_x and w_y), while a calibration curve was constructed to determine the *z*-coordinate. The AuNRs were sparsely immobilized in agarose gel and scanned along the *z*-direction at 10 nm intervals. The acquired images were fitted using an elliptical Gaussian function:

$$I(x,y) = I_0 + A\exp\left[-\frac{1}{2}\left[\frac{(x-x_0)^2}{{w_x}^2} + \frac{(y-y_0)^2}{{w_y}^2}\right]\right], \quad (1)$$

where I_0 is the background, A is the amplitude, x_0 and y_0 are the centroid coordinates of the AuNR, and w_x and w_y are the image widths in the *x*- and *y*-directions, respectively.

For localization in the *z*-direction, the w_x and w_y values were compared with those of the calibration curve to find the best-matched *z*-coordinates, *i.e.*, those with the minimal distance (D):

$$D = \sqrt{\left(w_x^{1/2} - w_{x,cal}^{1/2}\right)^2 + \left(w_y^{1/2} - w_{y,cal}^{1/2}\right)^2}. \quad (2)$$

A combination of the above procedures allowed us to superlocalize the probe in three dimensions and construct the corresponding tracking trajectory.

The rotational angles (φ and θ) of the AuNRs during SPT imaging were determined according to changes in the TSPR and LSPR scattering intensities, respectively. The above scattering intensities were normalized and fitted as follows:

$$I_{\mathrm{Nor.TSPR}} = \cos^2\varphi, \qquad (3)$$

$$I_{\mathrm{Nor.LSPR}} = \sin^2\theta, \qquad (4)$$

where $I_{\mathrm{Nor.\ TSPR}}$ and $I_{\mathrm{Nor.\ LSPR}}$ are the normalized intensities of the TSPR and LSPR signals, respectively, for the AuNRs after background subtraction.

Analysis of the instantaneous translational speed during the entire single-particle tracking required knowledge of the superlocalized *x*-, *y*-, and *z*-coordinates. The interval distance (d_{t}) between two contiguous coordinates was calculated as follows:

$$d_{\mathrm{t}} = \sqrt{(x_{\mathrm{t+1}} - x_{\mathrm{t}})^2 + (y_{\mathrm{t+1}} - y_{\mathrm{t}})^2 + (z_{\mathrm{t+1}} - z_{\mathrm{t}})^2}, \qquad (5)$$

where (x_{t}, y_{t}, z_{t}) and ($x_{\mathrm{t+1}}$, $y_{\mathrm{t+1}}$, $z_{\mathrm{t+1}}$) represent the two contiguous coordinates of the tracking trajectory of a given AuNR. The instantaneous translational speed (v_{t}) was then calculated as follows:

$$v_{\mathrm{t}} = \frac{d_{\mathrm{t}}}{t}, \qquad (6)$$

where *t* is the time interval between two contiguous frames.

The MSD was calculated based on the 3D reconstructed trajectory to determine the motion type of a single particle and to analyze the particle dynamic information in a quantitative manner. The MSD was defined as follows:

$$MSD(n\Delta t) = \frac{1}{N-n}\sum_{i=1}^{N-n}[(x_{i+n} - x_i)^2 + (y_{i+n} - y_i)^2 + (z_{i+n} - z_i)^2] \qquad (7)$$

where $N-n$ is the corresponding number of displacements, Δt is the interval time between two contiguous frames, (x_{i}, y_{i}, z_{i}) are the starting particle coordinates, and ($x_{\mathrm{i+n}}$, $y_{\mathrm{i+n}}$, $z_{\mathrm{i+n}}$) are the particle coordinates at a time interval of $n\Delta t$.

For 3D Brownian diffusion, the MSD showed a linear relationship with the lag time (t_{lag}). D_c represents the 3D diffusion coefficient, which could be calculated from the MSD plot slope as follows:

$$MSD = 6D_c t_{lag} \qquad (8)$$

Fitting the MSD with the formula allowed us to determine the motion types of AuNR diffusion, as well as the relevant parameters, such as the diffusion coefficient (D_c).

2 Supporting results and discussion

2.1 Functionalization of the AuNRs for single-particle tracking

The morphology, size, and optical/surface properties of native AuNRs, CTAB-AuNRs, and Fu-AuNRs were probed using TEM, UV-vis absorbance spectroscopy, FT-IR spectroscopy, and zeta potential measurements (Fig. S6). TEM imaging revealed that the three types of AuNRs exhibited a rod-like morphology (5 nm diameter × 15 nm length) and the Fu-AuNRs contained an outer shell composed of fucoidan (Fig. S6b). Furthermore, the UV-vis absorbance spectra revealed that the native AuNRs (I) exhibited a TSPR absorption peak at 513 nm and a LSPR absorption peak at 725 nm (Fig. S6c), leading to strong scattering signals under the appropriate illumination. The TSPR and LSPR absorption peaks of the Fu-AuNRs (III) were slightly red-shifted relative to those of the CTAB-AuNRs (II) owing to the presence of fucoidan (Fig. S6c). To confirm the conjugation of fucoidan to the AuNRs, we compared the FT-IR spectra of the native AuNRs (I), CTAB-AuNRs (II), and Fu-AuNRs (III) (Fig. S6d). The spectra of the CTAB-AuNRs (II) and Fu-AuNRs (III) exhibited a characteristic peak at ~1465 cm^{-1}, which was ascribed to the $-N(CH_3)_3^+$ group of CTAB. The peaks at ~1220 and ~840 cm^{-1} observed for the Fu-AuNRs were assigned to the S=O asymmetric stretching and C−O−S stretching vibrations of the sulfate groups, respectively, thereby confirming the successful conjugation of fucoidan to the AuNR surface. Moreover, the originally negative zeta potential of the native AuNRs (−12.5 mV, I) became positive following modification with CTAB (+37.5 mV for the CTAB-AuNRs, II) but became negative once more following conjugation with fucoidan (−24.7 mV for Fu-AuNR, III) (Fig. S6e).

3 Supporting figures

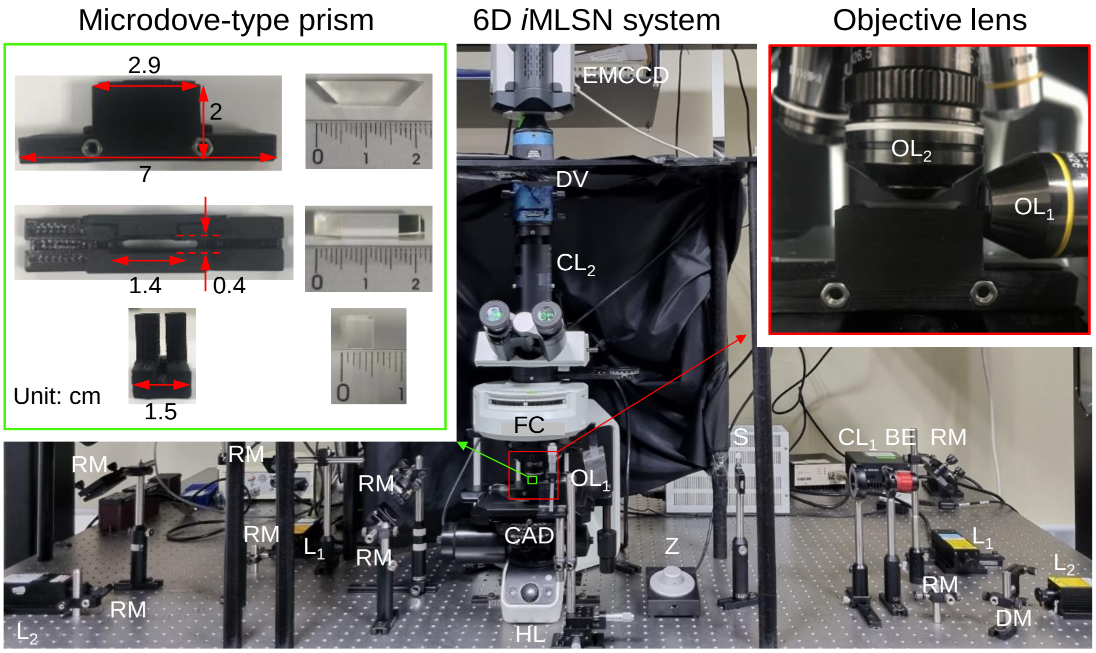

Fig. S1 Physical layout of the 6D *i*MLSN system for real-time spatiotemporal SPT imaging. (Left green box) Photograph of the microdove-type prism and holder. (Right red box) Photograph of the microdove-type prism and holder on a stage with detection and illumination objective lenses. A polished microdove-type prism was used to fasten the cell coverslip (Left green box), and the polarized lasers were focused via optics to achieve TIR and light-sheet (LS) illumination on the left and right sides, respectively, of the microscope. DIC imaging from the bottom of the microscope was achieved through halogen lamp illumination.

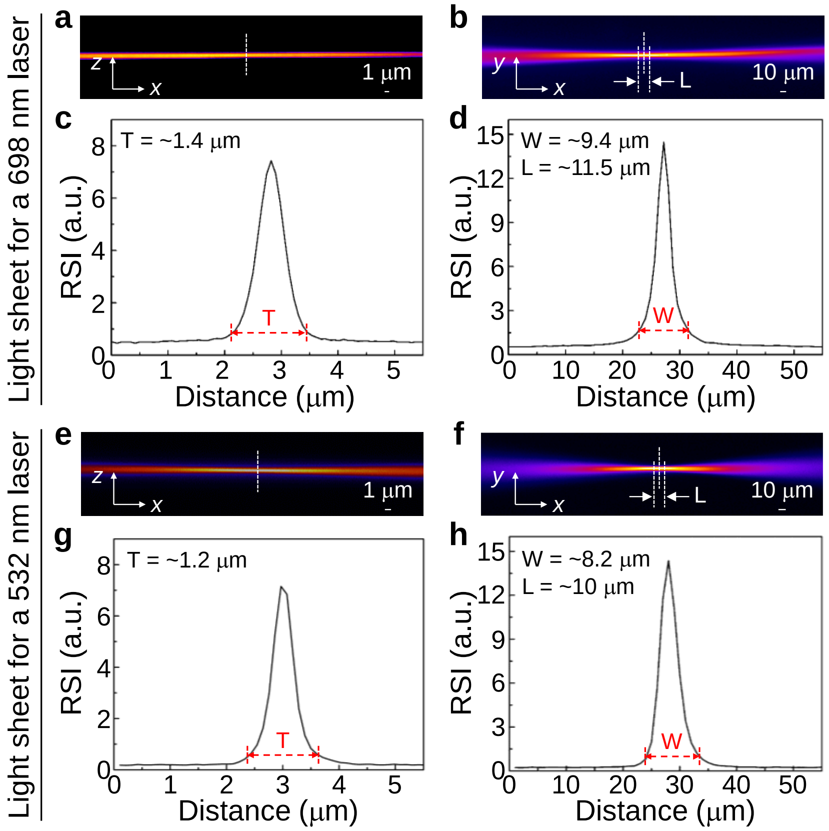

Fig. S2 Characterization of the 698 and 532 nm light sheets used in the *i*MLSN system. **a** and **b** Images of the 698 nm light sheet in the (**a**) *xz* and (**b**) *xy* planes. **c** and **d** Corresponding intensity plots along the white dotted lines shown in (**a**) and (**b**). **e** and **f** Images of the 532 nm light sheet in the (**e**) *xz* and (**f**) *xy* planes. **g** and **h** Corresponding intensity plots along the white dotted lines shown in (**e**) and (**f**). *T* = thickness, *W* = width, *L* = length.

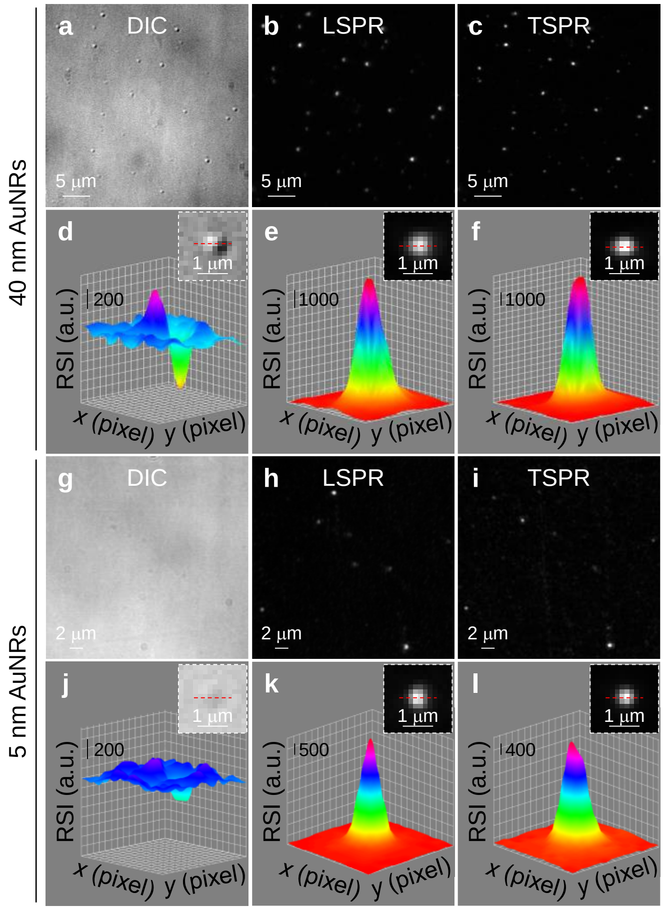

Fig. S3 Detection of different-sized AuNRs using the *i*MLSN system. **a** DIC, **b** LSPR scattering, and **c** TSPR scattering images of 40 nm native AuNRs on a PLL-coated coverslip. **d–f** Corresponding 3D intensity plots for the images shown in (**a–c**). **g** DIC, **h** LSPR scattering, and **i** TSPR scattering images of 5 nm native AuNRs on a PLL-coated coverslip. **j–l** Corresponding 3D intensity plots for the images shown in (**g–i**).

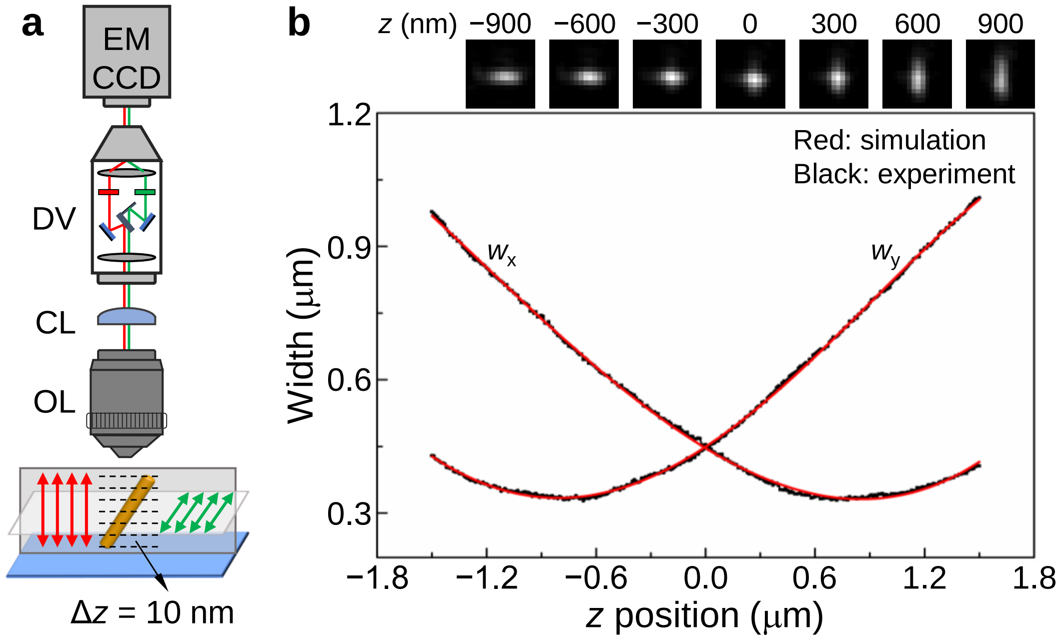

Fig. S4 Calibration curve of the point spread function widths obtained by scanning the immobilized Fu-AuNRs. **a** Schematic of the *i*MLSN setup used to generate a calibration curve for 5 nm Fu-AuNRs. **b** (top) PSF image of individual Fu-AuNRs at various *z*-positions. (bottom) Calibration curve showing the PSF widths w_x and w_y as a function of z derived from the *z*-sectioning of a Fu-AuNR at 10 nm intervals. The curve was plotted using average values obtained from seven Fu-AuNRs.

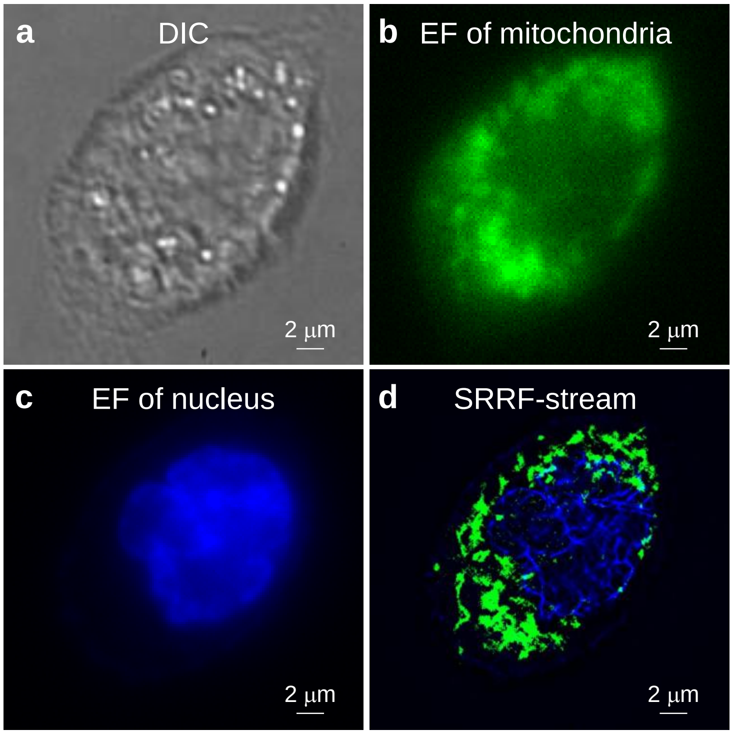

Fig. S5 Morphology and fluorescence images of stained A549 single live cells. **a** DIC image of a live A549 cell. **b** and **c** Epifluorescence (EF) images of the mitochondria (green) and nucleus (blue) of live A549 cells. **d** EF-based super-resolution radial fluctuation (SRRF-stream) image of a single live A549 cell with a stained nucleus and mitochondria.

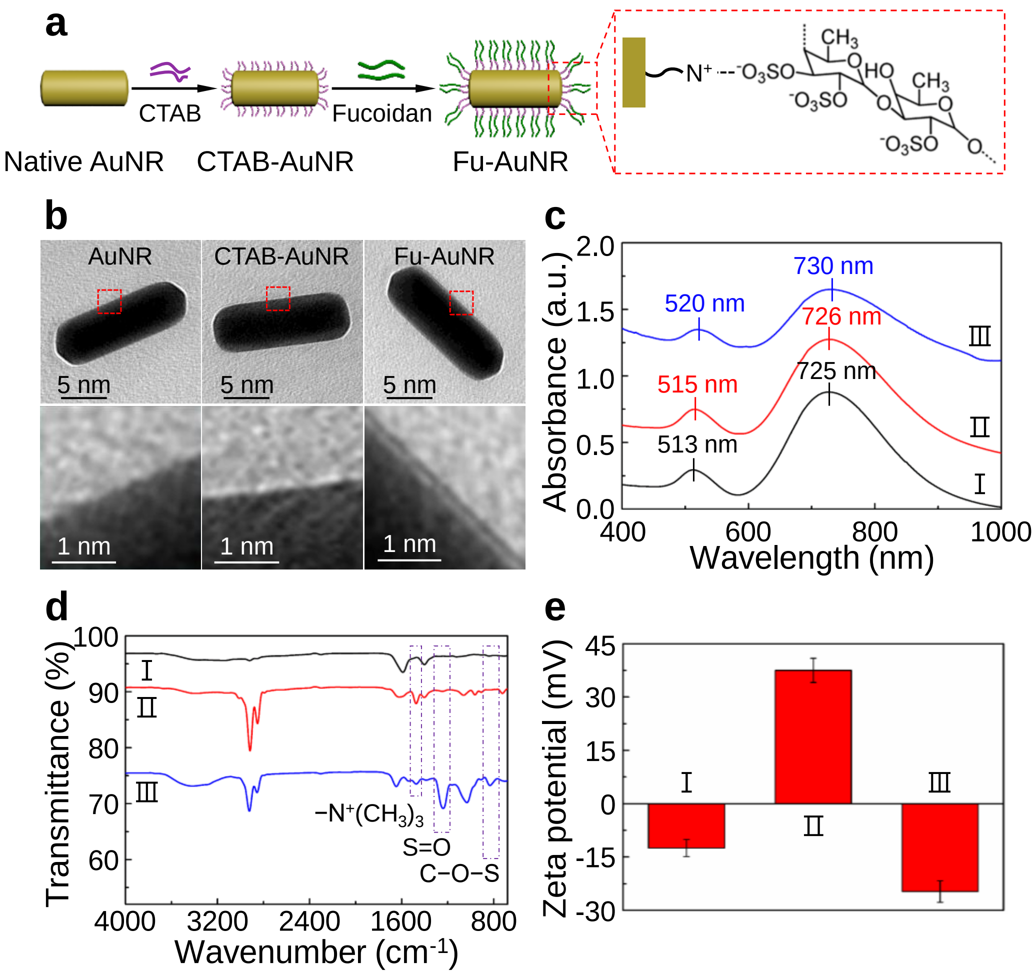

Fig. S6 Functionalization of the AuNRs for single-particle tracking. **a** Schematic outlining the preparation of the fucoidan-functionalized AuNRs. **b** TEM images of native AuNRs (5 nm diameter × 15 nm length), CTAB-AuNRs, and Fu-AuNRs. The lower images show expansions of the areas indicated by the red dotted lines in the upper images. **c** UV-vis absorption spectra, **d** FT-IR spectra, and **e** zeta potentials of the native AuNRs (I), CTAB-AuNRs (II), and Fu-AuNRs (III).

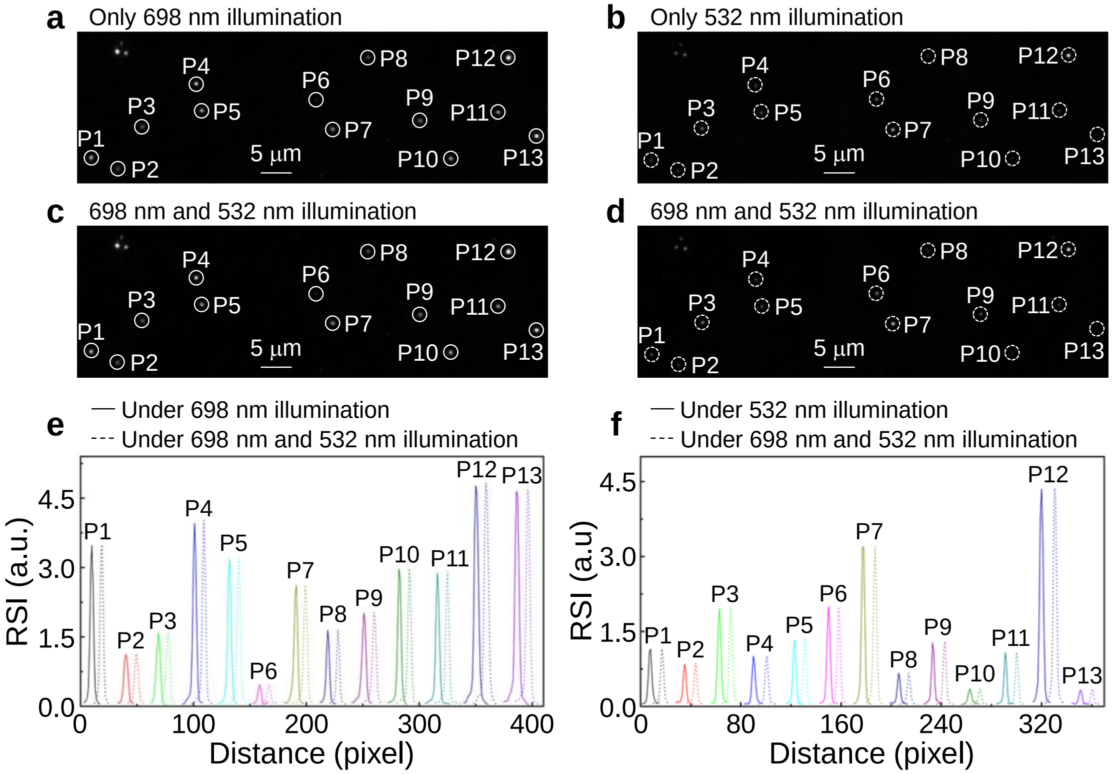

Fig. S7 Comparison of the scattering intensities of AuNRs in solidified PVA as determined by light-sheet illumination. **a** and **c** LSPR scattering of 5 nm native AuNRs embedded in solidified PVA under (**a**) 698 nm illumination only and under (**c**) 698 and 532 nm illumination. **b** and **d** TSPR scattering of 5 nm native AuNRs embedded in solidified PVA under (**b**) 532 nm illumination only and under (**d**) 698 and 532 nm illumination. **e** Comparison of the LSPR scattering intensities of the native AuNRs indicated in (**a**) and (**c**). **f** Comparison of the TSPR scattering intensities of the native AuNRs indicated in (**b**) and (**d**). The P number indicates the bright spots marked with white circles. RSI: relative scattering intensity.

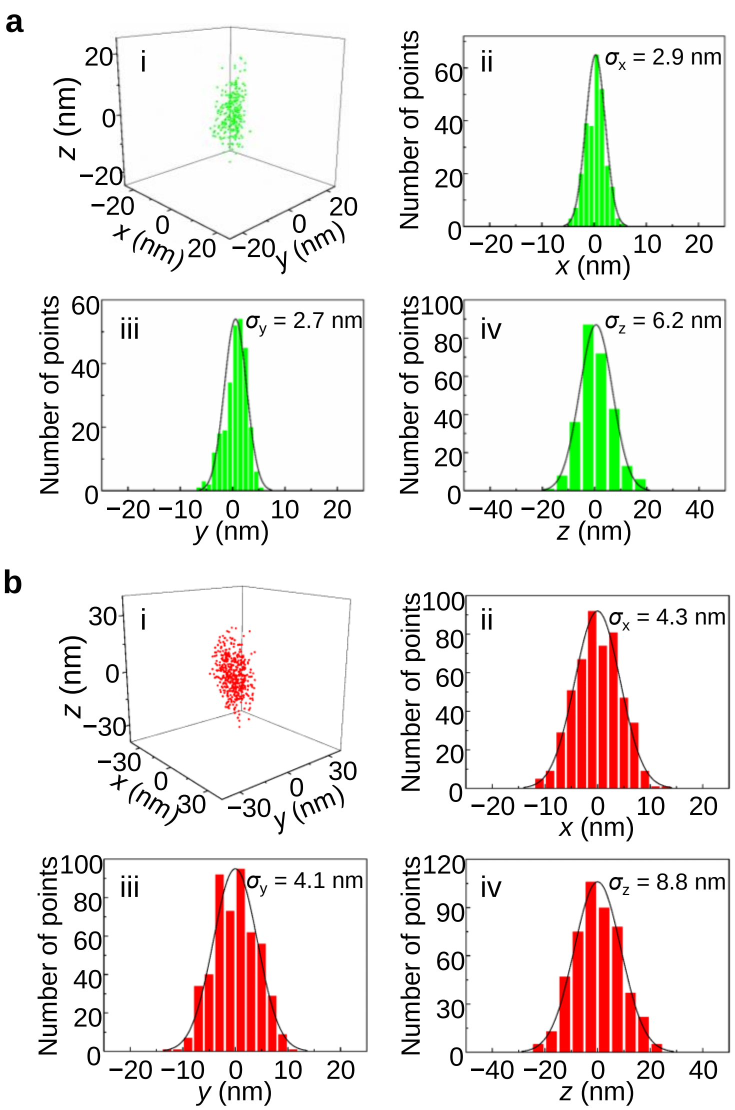

Fig. S8 3D Localization distributions of Fu-AuNRs on coverslip and in live cell as determined by *i*MLSN. (**i**) 3D distribution of the localization of 5 nm Fu-AuNRs **a** on coverslip and **b** in live cell. Histograms of the distributions along the (**ii**) *x*-, (**iii**) *y*-, and (**iv**) *z*-axes. σ indicates the standard deviation.

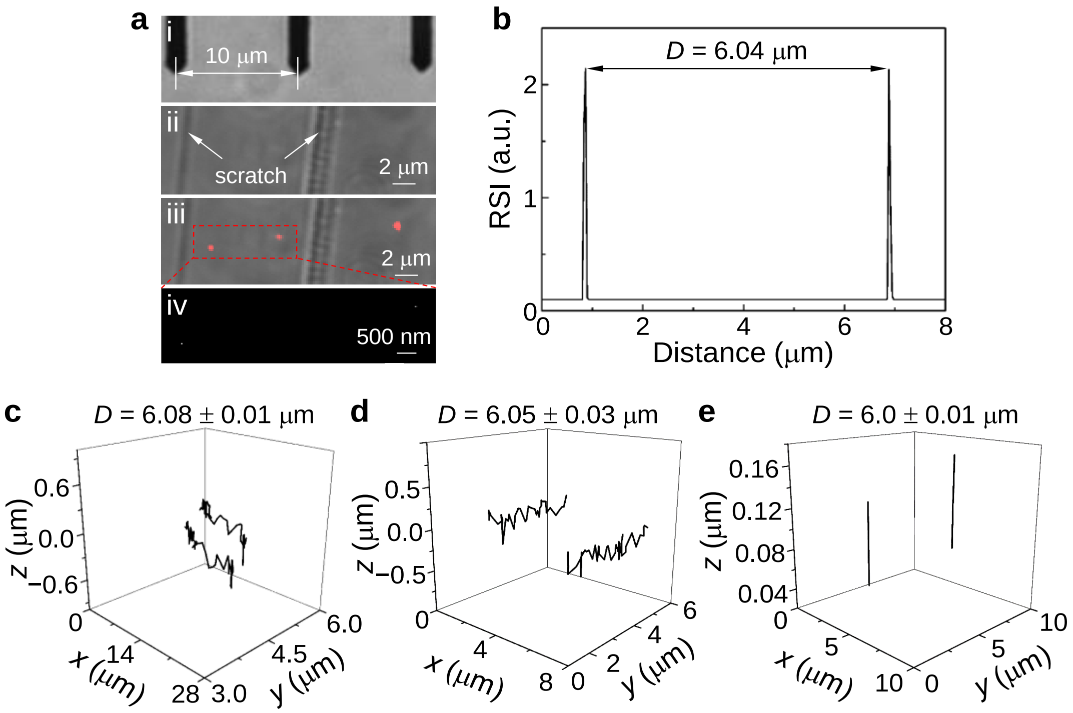

Fig. S9 Accuracy of the 6D *i*MLSN system for superlocalizing the positions of motorial Fu-AuNRs on a PLL-coated scratched coverslip. **a** (**i**) Calibration tails on a ruler for the distance calculations. (**ii**) DIC image of the scratched coverslip. (**iii**) Raw scattering image of the 5 nm Fu-AuNRs overlapped with the DIC image of the scratched coverslip. (**iv**) Reconstructed image of the two AuNRs shown in the red frame. **b** Plot of the reconstructed image of two AuNRs for distance calculations. **c**–**e** Single-particle tracking trajectory showing the stage motion in the (**c**) *x*-, (**d**) *y*-, and (**e**) *z*-directions. The mean *D* values ± standard deviations in (**c**–**e**) were averaged over 40 calculated distances during stage motion. *D* represents the distance between the two AuNRs in the red dotted frame shown in (**a**).

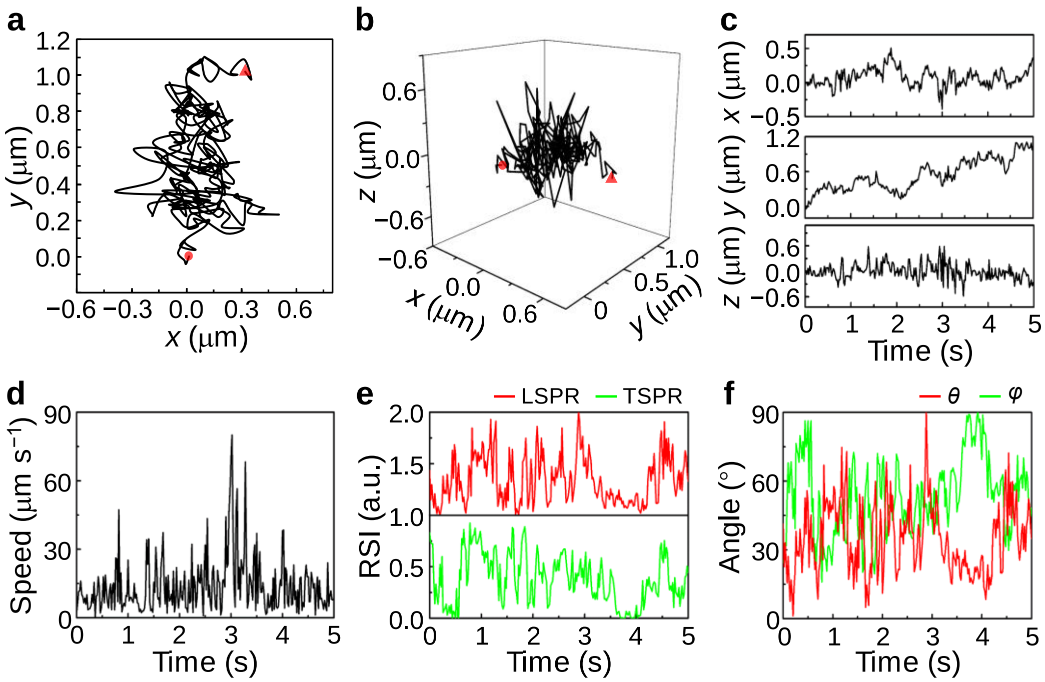

Fig. S10 Deciphering the real-time spatiotemporal SPT motion of a 5 nm native AuNR in 100% glycerol using 6D *i*MLSN. **a** 2D and **b** 3D trajectories of a single native AuNR in glycerol and **c** its corresponding displacements along the *x*-, *y*-, and *z*-axes. **d** Instantaneous speed, **e** normalized intensities, and **f** calculated angles of a single native AuNR in glycerol as a function of time. The red dot and triangle denote the movement start and end points, respectively.

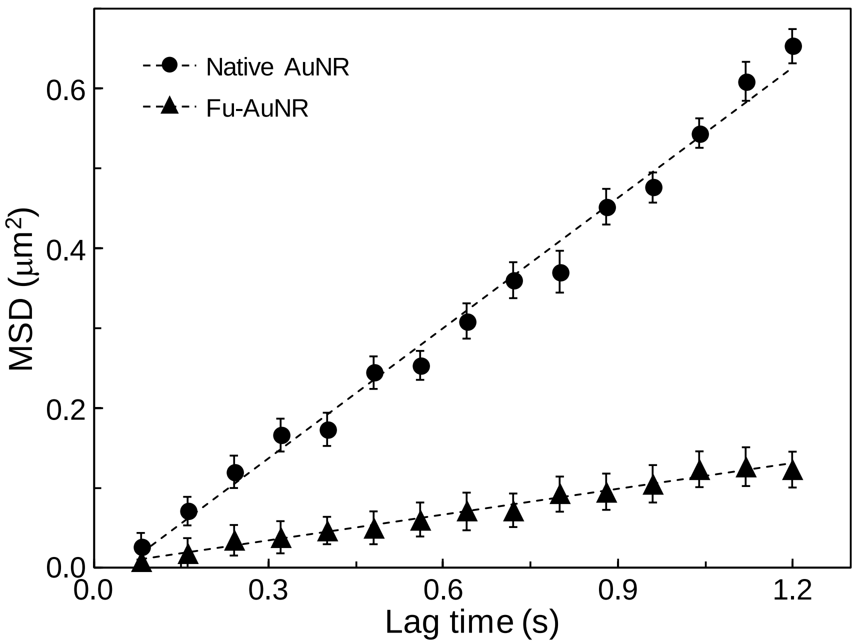

Fig. S11 Measured MSD values of various 5 nm AuNRs in 100% glycerol as a function of lag time. The corresponding diffusion coefficients (D_c) of the native AuNRs and Fu-AuNRs were 0.095 and 0.018 μm^2 s^{-1}, respectively.

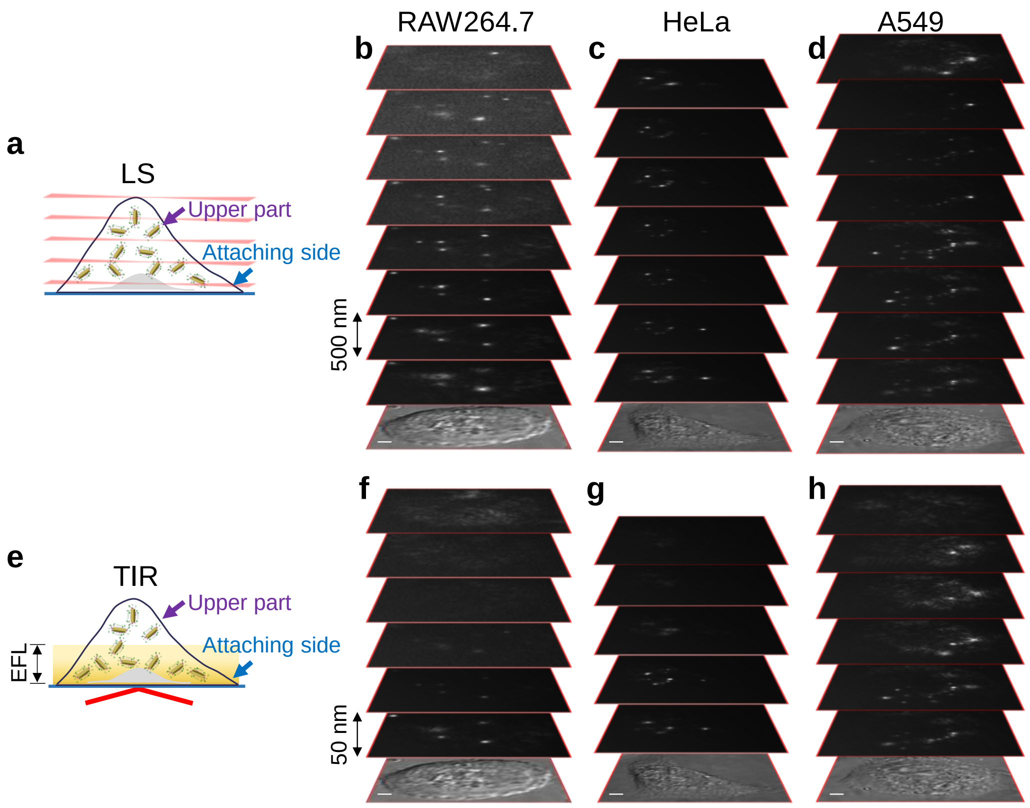

Fig. S12 Comparison of the TIR and LS microscopy results for the detection of the imaging probes in whole cells by optical *z*-sectioning with a thin light sheet. **a** and **e** Schematics of light-sheet (LS)-based and TIR scattering-based detection methods for Fu-AuNRs in live cells. **b**–**d** Light sheet-based *z*-sectioning images of the AuNRs in (**b**) RAW264.7, (**c**) HeLa, and (**d**) A549 cells. **f**–**h** TIR scattering-based *z*-sectioning images of the Fu-AuNRs in (**f**) RAW264.7, (**g**) HeLa, and (**h**) A549 cells. Both optical *z*-sectioning procedures employed a 10 nm interval. The scale bars in all images are 3 µm. The Fu-AuNRs were incubated with live cells (A549, RAW264.37, and HeLa cells) for 4 h and washed thrice with DPBS. The TIR and *i*MLSN techniques were subsequently used to detect the Fu-AuNRs in live cells by sectioning optically along the *z*-direction.

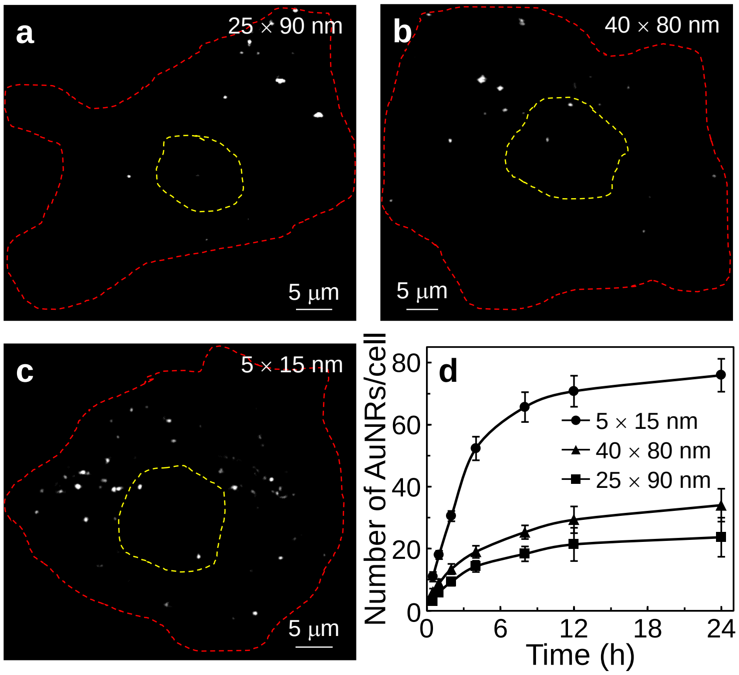

Fig. S13 Comparison of the uptake of 25 × 90 nm Fu-AuNR, 40 × 80 nm Fu-AuNR, and 5 × 15 nm Fu-AuNR of living A549 cells. Representative scattering images of **a** 25 × 90 nm Fu-AuNR, **b** 40 × 80 nm Fu-AuNR, and **c** 5 × 15 nm Fu-AuNR in living A549 cells after 2 h of incubation. **d** Number of Fu-AuNRs taken up by single A549 cells over time. The red- and yellow-dotted lines in (**a**–**c**) indicate the contours of the cell and nucleus, respectively.

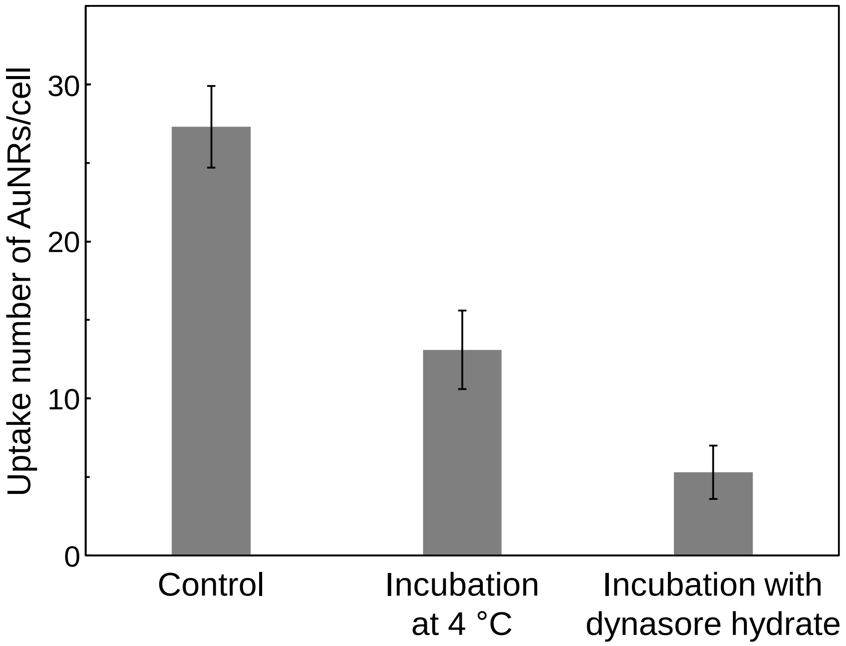

Fig. S14 Validation of receptor-mediated endocytosis by incubating A549 cells with Fu-AuNRs under normal conditions (37 °C and 5% CO_2, control), at a temperature of 4 °C, and under dynasore hydrate (80 µM) treatment. Comparison of the uptake of 5 nm Fu-AuNRs in live A549 cells incubated for 4 h under normal conditions (37 ℃ and 5% CO_2, left column), at 4 ℃ (middle column), and in the presence of dynasore hydrate (right column). Each result represents the average of the AuNR uptake number recorded for 21 cells (mean ± standard deviation, n = 21).

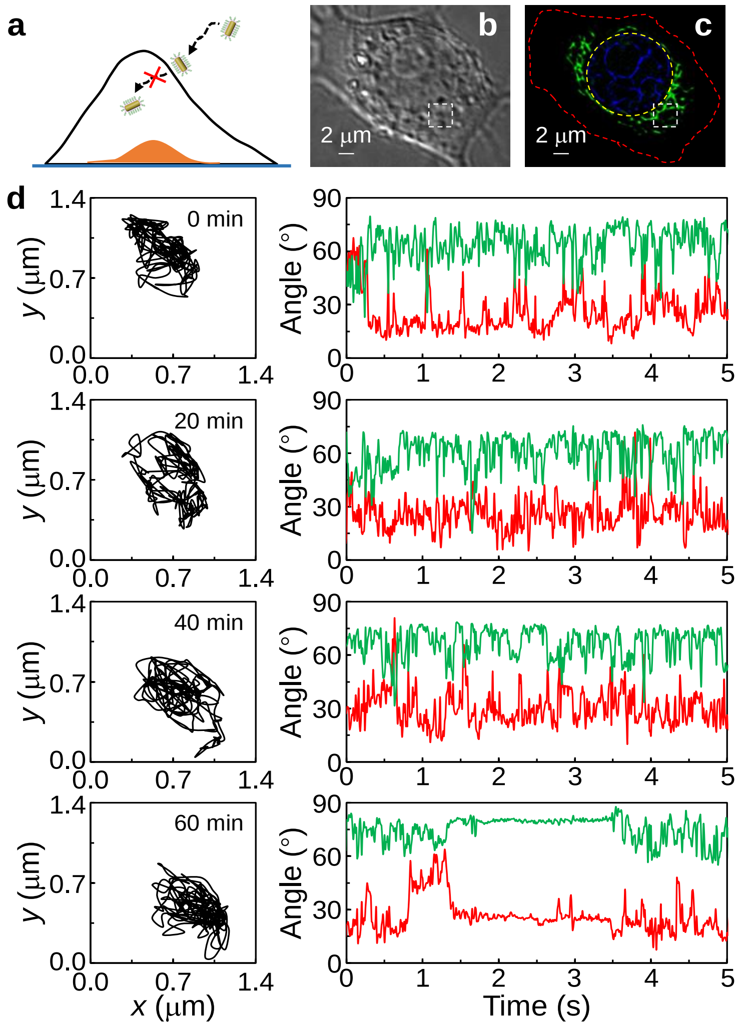

Fig. S15 Real-time visualization of endocytosis of 5 nm Fu-AuNRs on the upper membrane of live cells under dynasore treatment. **a** Schematic of real-time 5 nm Fu-AuNR tracking on the upper membrane of live A549 cells under dynasore treatment. **b** and **c** DIC and EF-based SRRF images of an A549 cell with a stained nucleus (blue) and mitochondria (green) after dynasore treatment. The red- and yellow-dotted lines in (**c**) indicate the contours of the cell and nucleus, respectively. **d** Tracking trajectories and rotation angles of the Fu-AuNRs on the upper membrane of live A549 cells highlighted in the white frame in (**b**) and (**c**) over 1 h.

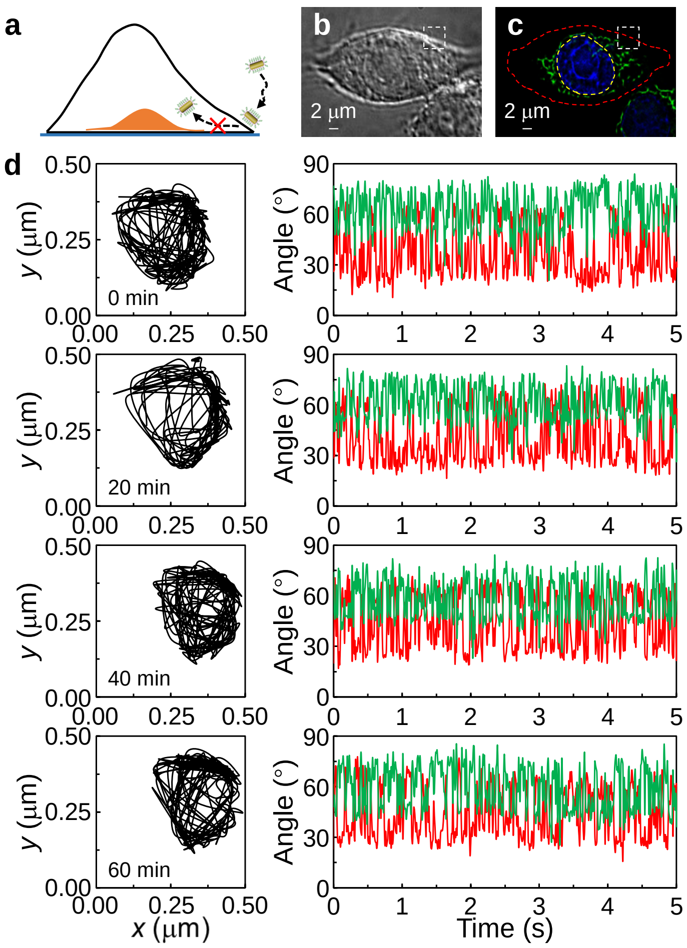

Fig. S16 Real-time visualization of endocytosis of 5 nm Fu-AuNRs on the attaching side membrane of live cells under dynasore treatment. **a** Schematic of real-time 5 nm Fu-AuNR tracking on the attaching side membrane of live A549 cells under dynasore treatment. **b** and **c** DIC and EF-based SRRF images of an A549 cell with a stained nucleus (blue) and mitochondria (green) after dynasore treatment. The red- and yellow-dotted lines in (**c**) indicate the contours of the cell and nucleus, respectively. **d** Tracking trajectories and rotation angles of Fu-AuNRs on the attaching side membrane of live A549 cells highlighted in the white frame in (**b**) and (**c**) over 1 h.

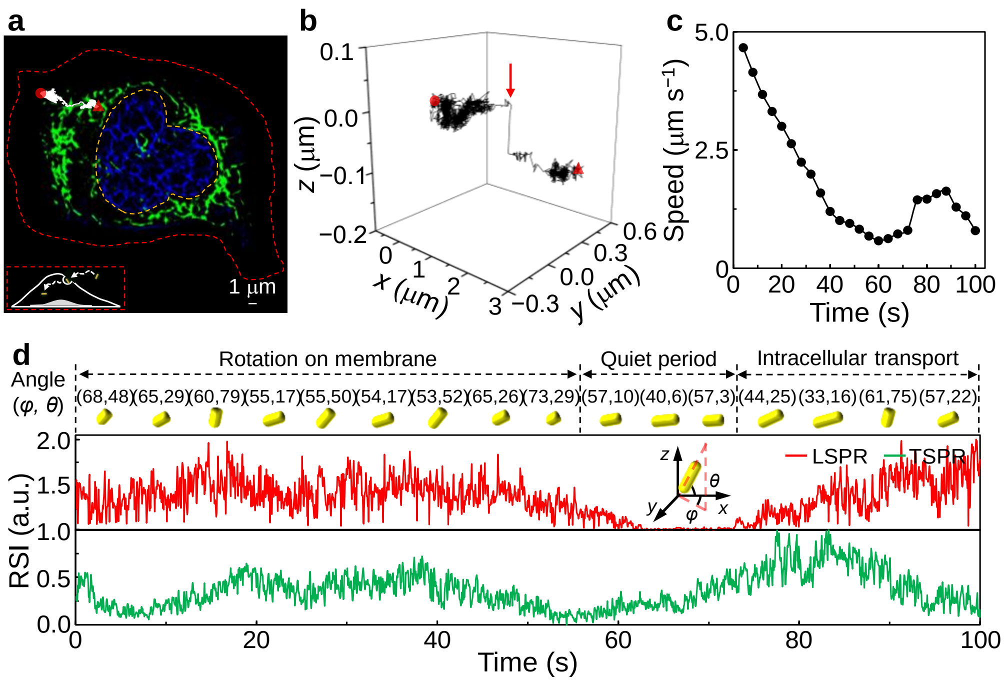

Fig. S17 Representative single 5 nm native AuNR 6D spatiotemporal tracking on the upper membrane of a single living A549 cell. **a** EF-based SRRF image of a single A549 cell with a stained nucleus (blue) and mitochondria (green). (Inset) 2D tracking trajectory of native AuNR indicated by the white line. The red- and yellow-dotted lines indicate the contours of the cell and nucleus, respectively. The red circle and triangle denote the start and end points of movement, respectively. **b** 3D tracking trajectory of a native AuNR highlighted in the white line in (**a**). The red arrow indicates the internalization of the native AuNR. **c** and **d** Corresponding speeds and descriptions of the different rotational states of a single native AuNR on the upper membrane of a single living A549 cell.

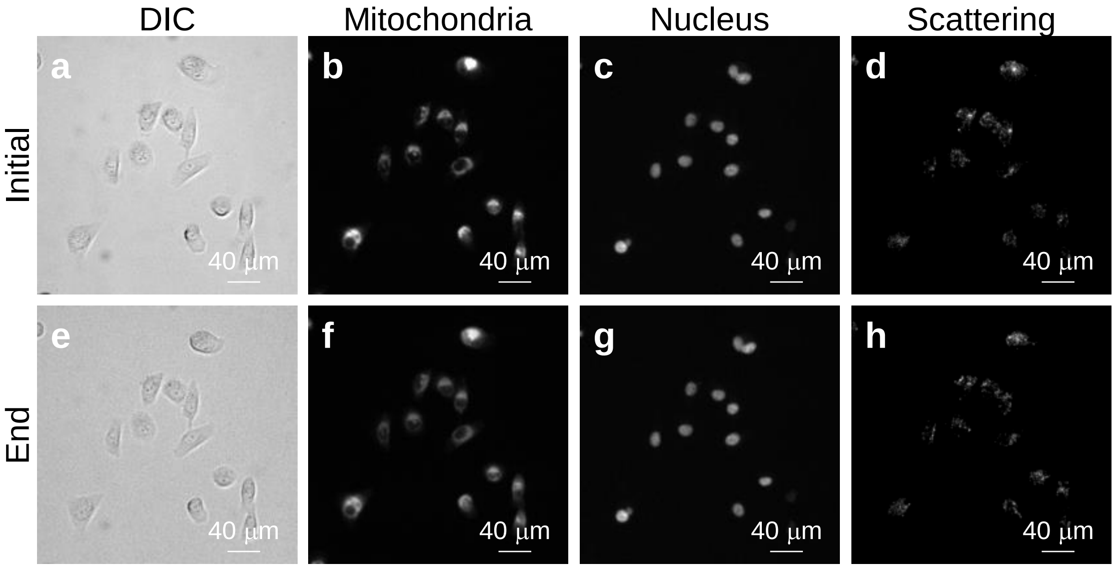

Fig. S18 Trypan blue assay for A549 cell viability measurements. **a, e** DIC images, **b, f** fluorescence images of mitochondria, **c, g** fluorescence images of nucleus and **d, h** scattering images of internalized Fu-AuNRs of A549 cells at (Top) initial and (Bottom) end stages of SPT imaging by *i*MLSN. Conditions: The live A549 cells were incubated with Fu-AuNRs and stained with fluorescence dyes. After SPT imaging, 0.4% trypan blue solution was added to the cell coverslip. We acquired their DIC, fluorescence, and scattering images at the initial and end stages of SPT imaging (~1 h).

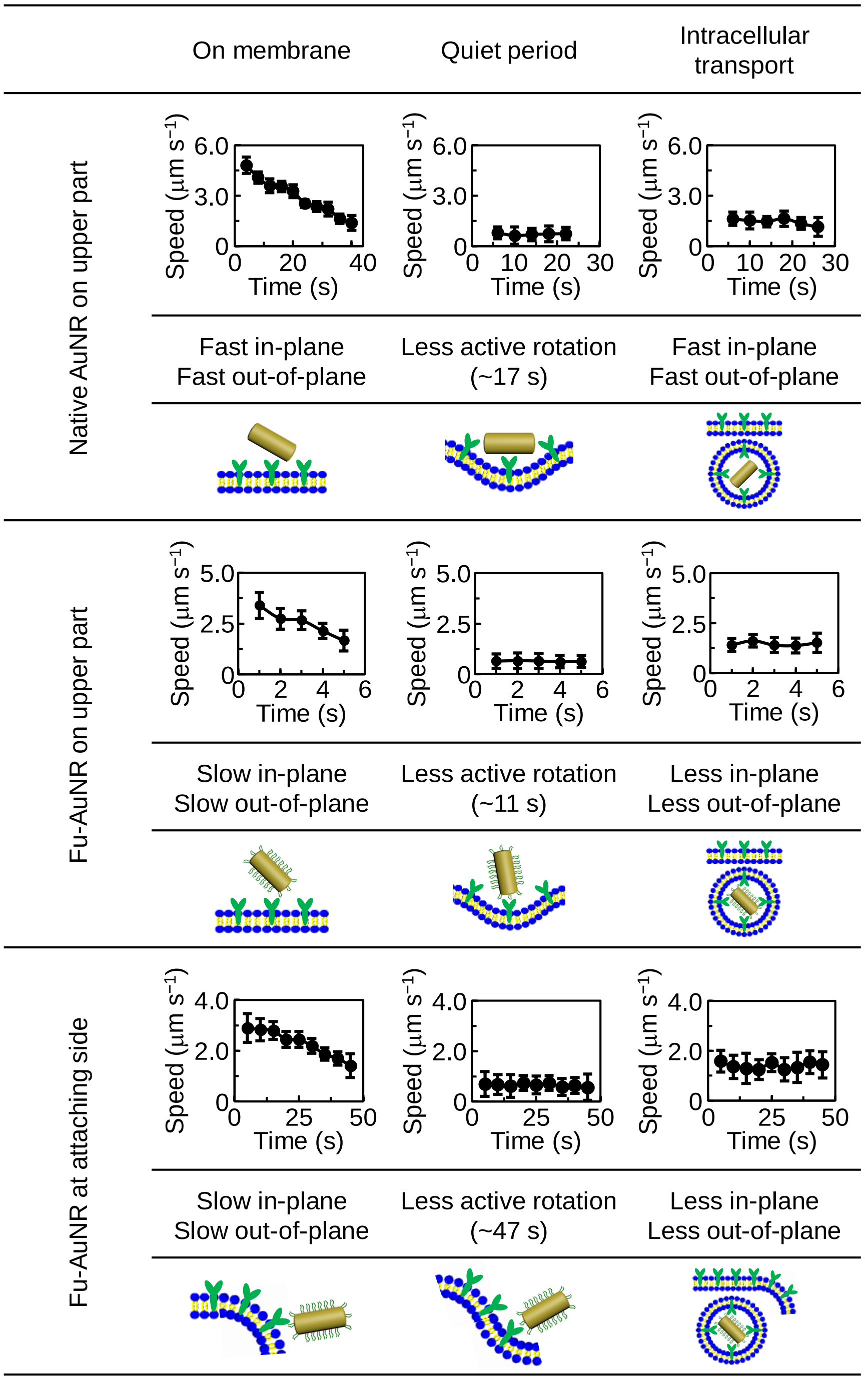

Fig. S19 Comparison of motion behavior of single 5 nm AuNRs tracking on the upper part and at the attaching side of single live A549 cells. These results used the average values obtained from five sets of SPT imaging, respectively.

Movie S1 Detection of native AuNRs (5 nm diameter × 15 nm length) in 100% glycerol by the *i*MLSM system. The AuNRs were simultaneously illuminated by 698 and 532 nm light sheets.

Movie S2 Motion and scattering intensity of Fu-AuNRs on a cell membrane at the upper part of a single live cell upon treatment with dynasore hydrate.

Movie S3 Motion and scattering intensity of Fu-AuNRs on a cell membrane at the attaching side of a single live cell upon treatment with dynasore hydrate.

Movie S4 (left) SPT imaging of 5 nm Fu-AuNRs on a cell membrane at the upper part of a single live A549 cell. (right) Corresponding changes in the scattering intensity of the 5 nm Fu-AuNRs in single live A549 cells for CH1 and CH2.

Movie S5 (left) SPT imaging of 5 nm Fu-AuNRs on a cell membrane at the attaching side of a single live A549 cell. (right) Corresponding changes in the scattering intensity of the 5 nm Fu-AuNRs in single live A549 cells for CH1 and CH2.
